# A phage-selective trigger hints at an SOS-independent mechanism of prophage induction by oxidative stress

**DOI:** 10.1039/d5sc04923g

**Published:** 2025-11-11

**Authors:** Magdalena Jancheva, Thi-Hong Nhung Nguyen, Felix Anderl, Shubham Joge, Jessica Neubauer, Clarissa Rominger-Baumann, Alexandra Walter, Golo Storch, Thomas Böttcher

**Affiliations:** a University of Vienna, Faculty of Chemistry, Institute for Biological Chemistry & Centre for Microbiology and Environmental Systems Science, Department of Microbiology and Ecosystems Science Josef-Holaubek-Platz 2 (UZA II) 1090 Vienna Austria thomas.boettcher@univie.ac.at; b Technical University of Munich (TUM), School of Natural Sciences and Catalysis Research Center (CRC) Lichtenbergstraße 4 85747 Garching Germany

## Abstract

We identified a prophage-selective induction in poly-lysogenic *Staphylococcus aureus* induced by certain phenazines, such as pyocyanin, produced by *Pseudomonas aeruginosa*. Using a focused library of phenazines, we discovered that prophage induction correlates not with antibiotic activity but with the compounds' ability to undergo redox cycling and generate reactive oxygen species (ROS). Importantly, we demonstrated that strong oxidative agents alone could selectively induce one of multiple prophages harbored by the bacterial host. Based on these findings, we propose the High-Level Oxidative Stress (HiLOS) response as an SOS-independent mechanism of prophage induction.

## Abstract 2

We report an SOS-independent mechanism of selective prophage induction in a poly-lysogenic *Staphylococcus aureus* host triggered by redox cycling of phenazine compounds.

## Graphical abstract

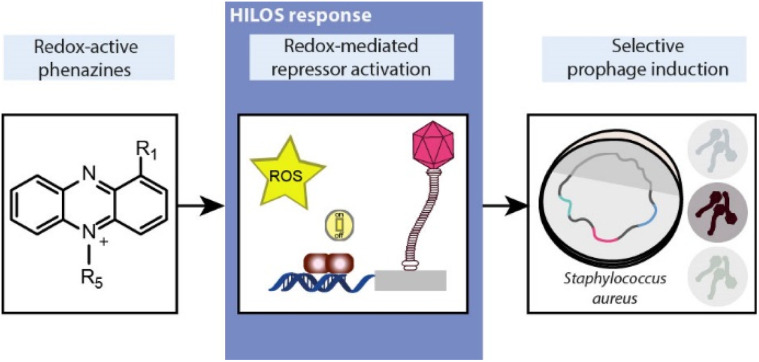

Phages are viruses that have co-evolved with their bacterial hosts for millions of years and play important roles in all microbial environments.^1^ Their enormous abundance in the human gut helps to maintain intestinal balance and at the same time protects against pathogen colonization.^2^ While virulent phages are natural foes of bacteria,^3^ temperate (lysogenic) phages are beneficial for their hosts as they encode virulence factors, serve as weapons against competitors, and provide immunity against infections of other phages.^4^ These phages reside as prophages within the genomes of their respective host bacteria and constitute a substantial fraction of the population of free phage particles in the human gut.^5^ Most bacteria are suggested to carry at least one prophage, making temperate phages highly prevalent in the human microbiome.^6^

Prophages can be induced stochastically^7^ or by specific stimuli to resume a lytic lifestyle. This lysis–lysogeny switch is central for controlling bacteria–phage interactions and thereby modulating their tripartite relationship with the human host.^8^ The lysis–lysogeny decision is typically regulated by the SOS response, which is triggered by DNA damage caused for example by alkylating agents, certain antibiotics and other distinctive small molecules.^9,10^ SOS response-driven prophage induction is also suspected to play a role in the human gut microbiome *via* the bacterial toxin colibactin.^11^

We have recently discovered that pyocyanin, a phenazine compound produced by *Pseudomonas aeruginosa*, selectively induces a single prophage in a poly-lysogenic *Staphylococcus aureus* host.^12^ In contrast, the DNA-alkylating agent mitomycin C, a general SOS-response trigger, induces all prophages in this strain, suggesting that pyocyanin operates through a distinct mechanism. Prophage induction beyond the SOS response is poorly understood, especially regarding the recently discovered phenomenon of selective prophage induction. Herein, we aim to comprehensively elucidate the mechanism of prophage induction by phenazines using a synthetic library and a detailed biochemical characterization.

In order to gain insights into the structural characteristics required for prophage induction, we examined the chemical space of phenazines beyond pyocyanin. To this aim, we synthesized a focused library of 13 phenazines and combined them with commercially available compounds, resulting in a total of 20 compounds with various substitutions in the 2-, 4-, 5-, 7-, and 8-positions. The synthetic routes and structures of these phenazines are depicted in Fig. 1a and S1. We adopted the synthesis strategy of Garrison *et al.*^13^ involving the oxidation of 3-methoxycatechol with *o*-chloranil to 3-methoxycyclohexa-3,5-diene-1,2-dione and subsequent condensation with *o*-phenylenediamine and derivatives harboring the respective residues at 7- and 8-position to obtain compounds 1, 6 and 7. Compound 1 was demethylated using boron tribromide (BBr_3_) to afford 1-hydroxyphenazine (8) or was substituted at 4- or 2,4-positions by reacting with corresponding *N*-halosuccinimide to obtain halogenated 1-methoxy phenazines (2–5). Similarly, demethylation of the 4-bromo-1-methoxyphenazine (2) with BBr_3_ yielded compound 10. The 1-hydroxyphenazine (8) was brominated using NBS to obtain compound 9, which was esterified with different acyl chlorides yielding the respective 2,4-dibromo phenazine esters (11–13). The quaternization of compound 2 or acridine with dimethyl sulfate gave the corresponding methylated derivatives 19 and 20. Compounds 14–18 were obtained commercially.

**Fig. 1:**
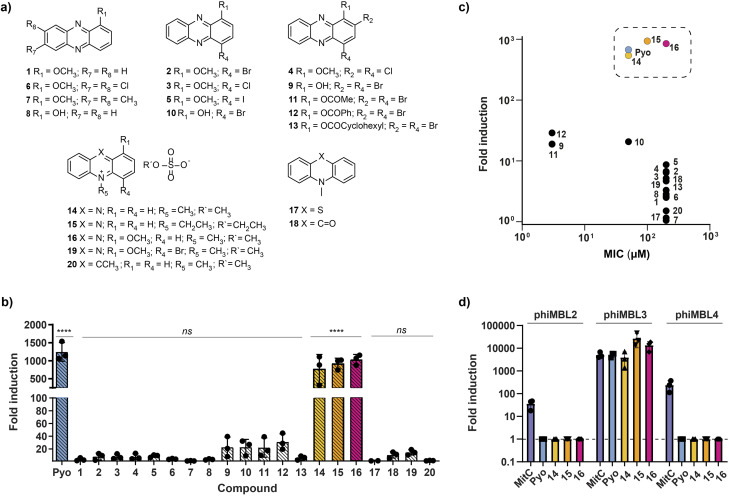
(a) Structures of the phenazine derivatives tested for prophage induction in *S. aureus* ATCC 6341. (b) Pyocyanin and compounds 1–20 trigger virion production in *S. aureus* with different intensity levels. Fold induction relative to the untreated control is shown for the highest inducing concentrations of each compound (100 µM (1–8, 13, 16–20), 50 µM (15), 25 µM (Pyo, 10, 14) and 1.5 µM (9, 11, 12)). (c) Correlation between the MIC values and fold induction of the respective phenazine derivatives. (d) qPCR-based fold induction for prophage-active phenazines and mitomycin C at their highest inducing concentrations (MitC: 1.5 µM, Pyo and 14: 25 µM, 15: 50 µM and 16: 100 µM) relative to a DMSO control. Since no amplification was detected for any of the capsid genes in the DMSO-treated samples, a *C*_q_ value of 40 (the maximum number of qPCR cycles) was assigned to each. For (b and d), three independent biological replicates were performed for each compound at the respective concentrations. The mean values and corresponding standard deviations are provided. For (b) the data was analyzed with one-way ANOVA, followed by Dunnett's comparison test. ****, *P* < 0.0001; ns, not significant *vs.* DMSO control.

With this focused library, we aimed to investigate prophage induction and assess whether any observed activity correlates with antimicrobial efficacy. Given the potent antimicrobial properties of phenazines, we initially determined their minimum inhibitory concentration (MIC) values against our prophage-containing *S. aureus* strain. The 2,4-dibrominated phenazines 9, 11 and 12, exhibited the lowest MIC values of 3 µM. Compounds 10 and 14–16 also inhibited the growth of the strain between 50 and 200 µM, whereas all other phenazines showed no inhibition even at the highest tested concentration of 200 µM (Fig. S2). Next, we investigated the ability of each of the phenazines to induce prophages. Accordingly, *S. aureus* ATCC 6341 was treated with different concentrations of the compounds, starting either with 200 µM or the corresponding MIC value. The supernatants were collected, and the phage virions produced were quantified as plaque forming units (PFUs) using a double agar overlay assay on the *S. aureus* RN4220 indicator strain. Only three of the twenty phenazine compounds – 14, 15 and 16 – achieved phage induction levels comparable to the three orders of magnitude seen with pyocyanin. In comparison, compounds 9, 11 and 12, which exhibited the lowest MIC values, along with compound 10, only led to a 20- to 30-fold increase in PFU. The remaining phenazines, specifically compounds 1–8, 13 and 17–20, could trigger only a 2- to 10-fold increase in virion production (Fig. 1b and S3). The common motif among potent inducers was an alkylated, and thus charged, nitrogen on the phenazine ring. Our results further show that the phenazines with the lowest MIC values were not the most potent inducers suggesting that prophage induction by phenazines does not correlate with their antibiotic activity (Fig. 1c).

*S. aureus* ATCC 6341 is a poly-lysogenic host containing six prophage-like regions (PLRs), three of which are complete prophages (phiMBL2, phiMBL3 and phiMBL4).^12^ Having previously identified pyocyanin as the first prophage-selective inducer, we now aimed to examine selectivity on prophage level for the newly discovered potent inducers (compounds 14–16). To this aim we developed a qPCR method targeting capsid genes of the distinctive prophages. In brief, following bacterial host induction, the phage-containing supernatants were diluted, treated with DNase I to eliminate host DNA, and subjected to a heating step to disassemble the capsid proteins. The phage DNA was then amplified with sequence-specific primers. As a quality control measure, we used the *gyrB* gene, which encodes for gyrase, to confirm the absence of bacterial DNA contamination in the samples. Consistent with our previous findings, mitomycin C induced all three prophages, while pyocyanin triggered only the production of phiMBL3. Like pyocyanin, phenazines 14–16 caused selective induction of phiMBL3. At the highest concentration, compounds 15 and 16 showed a 2- to 4-fold stronger inductive response than pyocyanin (Fig. 1d).

To ultimately confirm that the selective phenazine inducers operate *via* the same mode of action as pyocyanin, we tested their activity in a pyocyanin-resistant mutant (pyo^R^) harboring a mutated type II NADH:quinone oxidoreductase gene. Accordingly, PFU reductions of one to two orders of magnitude were observed for the pyo^R^ mutant treated with the prophage-inducing phenazines in comparison to the wild-type strain suggesting that the three new phenazines indeed operate *via* the same mechanism as pyocyanin (Fig. S4a).

We previously hypothesized that reactive oxygen species (ROS) generated by pyocyanin could be responsible for prophage induction.^12^ To test this, we investigated whether ROS production by other phenazines in our focused library correlates with their prophage-inducing activity. We hence quantified ROS levels in *S. aureus* ATCC 6341 generated by the most potent prophage-inducers (14–16) and the halogenated compounds which exhibited the highest antibiotic activity (9–12). Similar ROS levels were detected for pyocyanin and compounds 14–16, while compounds 9–12 showed lower ROS formation at the highest inducing concentrations (Fig. S4b). Diminished ROS production was also recorded in the pyo^R^ mutant upon pyocyanin exposure, re-affirming the role of the oxidoreductase in the ROS generation process (Fig. S4c).

The antimicrobial potency of phenazines is attributed to their redox cycling ability in the presence of reducing agents like NAD(P)H in the bacterial cells. Under aerobic conditions, redox-active phenazines generate ROS by transferring electrons from NAD(P)H to molecular oxygen (Fig. 2a).^14^ An experimental approach using NADP(H) UV/vis spectra was previously developed to demonstrate the redox cycling ability of pyocyanin *in vitro*.^15^

**Fig. 2:**
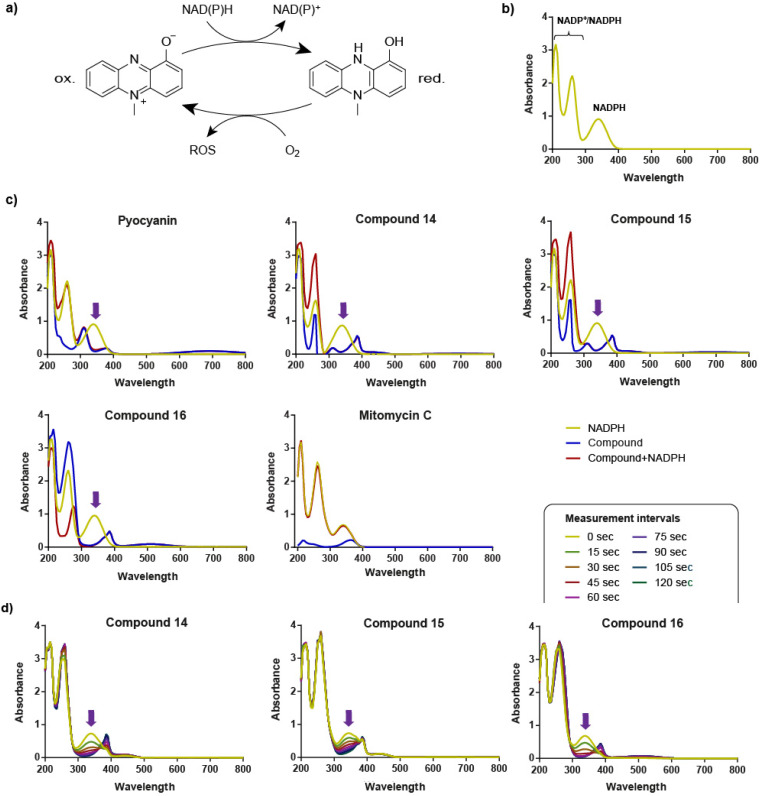
(a) Redox cycling of phenazines in the presence of NAD(P)H and O_2_. (b) Absorption spectrum of NADPH: the peak at 340 nm indicates the presence of reduced NADPH, while the peaks at 220 nm and 260 nm are characteristic of both the oxidized NADP^+^ and the reduced NADPH. (c) Absorption spectra of NADPH in phosphate buffer with pyocyanin, compounds 14–16, and mitomycin C. Reactions were monitored after 20 min incubation for redox-active phenazines and after 6 h incubation for mitomycin C. Yellow lines show the absorption spectrum of NADPH alone, blue lines represent the spectrum of the compound, and red lines show the spectrum of NADPH in the presence of a phenazine or mitomycin C. (d) Time-dependent oxidation of NADPH by compounds 14–16 over 120 seconds. Different colored lines represent measurements taken every 15 seconds. In (c and d) purple arrows indicate a decrease in NADPH-specific absorption at 340 nm.

When monitoring the spectra, three different wavelength peaks can be identified: at 220 nm for NAD(P)^+^, at 260 nm for the adenine moiety and at 340 nm for NADPH (Fig. 2b). Upon addition of a redox-active compound such as pyocyanin, the peak at 340 nm disappears as NADPH is fully oxidized.^16^ We adopted this approach to investigate the redox cycling ability of compounds 1–20 and mitomycin C using reaction times ranging from seconds to hours. As suspected, only the most potent prophage-inducing phenazines (14–16) and pyocyanin effectively oxidized NADPH after 20 min, as evidenced by the disappearance of the 340 nm peak (Fig. 2c). Time resolved absorbance measurements within short intervals of 15 s further established the strong redox cycling of these phenazines (Fig. 2d). In contrast, mitomycin C and phenazines that were inactive in prophage induction experiments did not result in significant decrease of the NADPH signal.

Compounds 9–12 oxidized NADPH at a marginal rate, while compound 19 only displayed a mild redox cycling ability after longer reaction times, likely insufficient to trigger potent prophage induction (Fig. S5). Finally, as ROS are generated during the reoxidation of reduced pyocyanin, we quantified the resulting hydroperoxide levels using the ferrous oxidation-xylenol orange (FOX) assay.^17^ Supporting our hypothesis, hydroperoxide was detectable with pyocyanin and the three potent prophage inducers (14–16), but not with the other phenazines or mitomycin C. Compound 19, which only exhibited mild redox cycling, also generated low concentrations of hydroperoxide (Table S1).

To assess the impact of the prophage-activating phenazines on the redox status of live cells, we measured the NAD^+^/NADH ratio in the wild-type strain and the pyo^R^ mutant (Fig. S6). Pyocyanin drastically decreased the ratio in the wild-type strain, while no such effect was observed in the pyo^R^ mutant, which already exhibited a lower basal ratio. These results further prove that the oxidoreductase is involved in pyocyanin mediated redox cycling.

We continued our studies with cyclic voltammetry (CV) measurements of the tested compounds to gain deeper insights into their redox chemistry in solution (all data were obtained in CH_3_CN, see SI for details). The parent compound pyocyanin (Pyo) was reversibly reduced at *E*_1/2_(Pyo/Pyo˙^−^) = −0.88 V *vs.* SCE and exhibits a second reduction of Pyo˙^−^ to Pyo^2−^ at −1.66 V *vs.* SCE (Fig. S7). The structurally related 1-hydroxyphenazine (8), which lacks the *N*-methyl group, is reduced at a more negative potential and thus less readily than pyocyanin (Fig. S8). The prophage-inducing *N*-substituted phenazinium salts 14 and 15 revealed properties similar to pyocyanin, however, they are reduced at more positive potentials.^18^ Both are characterized by a first reversible reduction at *E*_1/2_(14^+^/14˙) = −0.09 V and *E*_1/2_(15^+^/15˙) = −0.10 V *vs.* SCE followed by a second reduction at *E*_1/2_(14˙/14^−^) = −1.07 V and *E*_1/2_(15˙/15^−^) = −1.07 V *vs.* SCE, respectively (Fig. S9 and S10). This is consistent with their fast reaction with NADPH and the observed rapid generation of ROS species. The methoxy substitution in phenazinium salt 16 led to only slightly more negative redox potentials (*E*_1/2_(16^+^/16˙) = −0.17 V and *E*_1/2_(16˙/16^−^) = −1.10 V *vs.* SCE), which still resulted in a highly active compound (Fig. S11). The structurally similar, yet inactive, carbon analog 20 shows irreversible reduction *E*_P_ = −0.43 V *vs.* SCE (Fig. S12), assigned to dimer formation after the initial reduction step.^19–21^ These findings led us to conclude that the sufficient stability of ROS-generating compounds during redox cycling is likely a key parameter.

Antioxidant systems that neutralize reactive oxygen species are an essential part of the bacterial defense network against oxidative stress. These systems consist of enzymes specialized in eliminating the distinctive radicals generated. Catalases, for instance, substantially eradicate hydrogen peroxide in the bacterial cell, while superoxide dismutases act against superoxide radicals.^22^ It is therefore uncertain whether a single pulse of exogenously added ROS agents can trigger prophage induction *in situ*.

In order to examine if different types of ROS, besides H_2_O_2_, or reactive nitrogen species (RNS) could selectively induce prophages or phiMBL3 in *S. aureus* ATCC 6341, we tested three different radical-generating compounds, namely *tert*-butyl hydroperoxide (*t*BuHP), cumene hydroperoxide (CuHP) and diethylamine nonoate (DEA-NONOate). Remarkably, unlike H_2_O_2_, the organic hydroperoxides *t*BuHP and CuHP induced prophages by approximately two orders of magnitude at 500 µM and 1 mM compared to solvent control. Similarly, the NO-releasing compound DEA-NONOate showed comparable potency at 10 mM. With increasing cell toxicity, further increase of concentrations to 5 mM and 10 mM led to reduced phage inducing activity for *t*BuHP and CuHP (Fig. S13). Although these ROS/RNS agents showed remarkable prophage induction at high concentrations, their maximum induction potential was 2 to 50 times lower than that of pyocyanin (Fig. 3a). This observation suggests that supplemented ROS/RNS agents are rapidly depleted and the continuous production of ROS/RNS by redox-cycling compounds, such as phenazines, is required for the most effective and potent prophage induction.

**Fig. 3:**
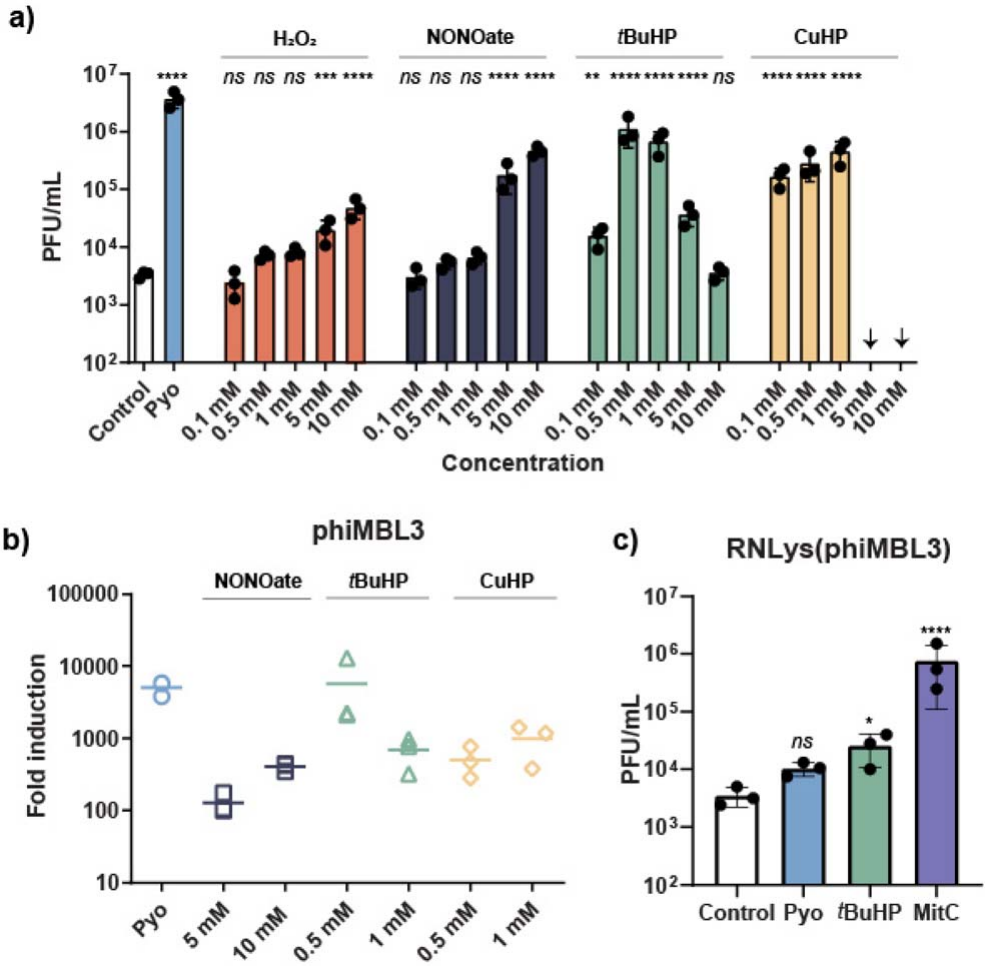
(a) Prophage induction by ROS/RNS agents (hydroperoxides and DEA-NONOate) in *S. aureus* ATCC 6341 in concentration dependence. Arrows indicate the absence of detectable PFUs. (b) qPCR-based fold induction for induced phiMBL3 with DEA-NONOate and hydroperoxides *t*BuHP and CuHP relative to a DMSO control. Because amplification of the capsid genes was not detected in the DMSO-treated samples, we assigned a *C*_q_ value of 40, corresponding to the total number of qPCR cycles, to each sample. (a and b) Pyocyanin (25 µM) served as the positive control. (c) PFU per mL counts for phiMBL3-lysogenized *S. aureus* RN4220 in the presence of pyocyanin (25 µM), *t*BuHP (1 mM) or mitomycin C (1.5 µM). For (a–c) all induction experiments, plaque assays, and qPCRs were performed in triplicates and the mean values with the corresponding standard deviations are reported. One-way ANOVA was performed for (b and c), followed by Dunnett's test. ****, *P* < 0.0001; ***, *P* < 0.001; **, *P* < 0.01; *, *P* < 0.05; ns, not significant *vs.* DMSO control.

Next, we aimed to investigate the selectivity of prophage induction by ROS/RNS agents using our previously established qPCR method. Gratifyingly, these ROS/RNS agents were highly prophage selective and induced only phiMBL3, as no amplification was detectable for phiMBL2 and phiMBL4 within 40 cycles. These results suggest that the prophage selectivity of pyocyanin and other phenazines is directly driven by the *in situ* generation of ROS/RNS through redox cycling. The lower induction potential observed with the plaque assay, compared to pyocyanin, was also confirmed using the qPCR method, reinforcing that a continuous supply of ROS/RNS species is essential for maximal prophage induction activity (Fig. 3b).

Additional experiments involving the pyo^R^ mutant showed that these compounds operate independently of the oxidoreductase, which appears to be essential for redox cycling in the presence of the phenazines. None or only a slight reduction in PFU counts was observed for pyo^R^ mutant treated with *t*BuHP and DEA-NONOate in comparison to wild-type *S. aureus* ATCC 6341, with exception to CuHP where no phage particles were detected in the pyo^R^-induced cells due to high cell toxicity (Fig. S14). The general sensitivity of pyo^R^ mutants to ROS/RNS agents leads us to conclude that induction by pyocyanin is a two-step process: first the generation of ROS/RNS followed by their downstream effect on activating the prophage. This first step can be bypassed by the direct addition of ROS/RNS agents in millimolar amounts.

Considering the fact that oxygen is required for ROS formation through pyocyanin redox cycling, we hypothesized that prophage induction would not occur under anaerobic conditions. *S. aureus* ATCC 6341 was not susceptible to pyocyanin when grown in the absence of oxygen at concentrations up to 200 µM. We therefore treated the cultures with pyocyanin (25 µM) or mitomycin C (1.5 µM) with the same concentrations used under aerobic conditions and quantified phage particles using a plaque assay. As expected, mitomycin C triggered phage production under both aerobic and anaerobic conditions, whereas pyocyanin failed to cause any prophage induction when host was grown anaerobically (Fig. S15).

Finally, we examined whether phiMBL3 itself could be induced in a strain with a genetic background, different from its native host. To test this, we lysogenized *S. aureus* RN4220, hereafter designated RNLys (phiMBL3) and determined its susceptibility to pyocyanin. As the lysogenized strain exhibited the same MIC value for the phenazine as the ATCC 6341 we exposed it to equivalent concentrations of mitomycin C, pyocyanin, or *t*BuHP. Mitomycin C strongly induced the prophage, whereas *t*BuHP and pyocyanin caused only minor or no induction suggesting that induction by pyocyanin and ROS agents differs mechanistically from SOS-mediated response triggered by mitomycin C and likely depends on the host genetic background (Fig. 3c). In comparison to ATCC 6341, RN4220 has several gene mutations in crucial genes affecting quorum sensing and virulence and it also lacks, apart from phiMBL2 and phiMBL4, the two staphylococcal pathogenicity islands (SaPIMBL1 and SaPIMBL6).^12^

Since substantial quantities of ROS/RNS (*i.e.* millimolar concentrations) are necessary for the selective induction of phiMBL3, we will here name this mechanistically distinct pathway of prophage induction a High-Level Oxidative Stress (HiLOS) response (Fig. 4). Prophage induction *via* the HiLOS response involves a truncated CI repressor^12^ and a strong oxidative pulse either by redox cycling agents or directly by strong ROS/RNS agents. The inducibility of phage phiMBL3 by MitC, despite its truncated CI repressor lacking a proteolytic active site, remains unclear. Notably, the truncated CI is encoded adjacent to a metalloprotease gene resembling ImmA/ImmR-like lysogeny systems, which are known to be SOS-induced.^23^ Furthermore, N-terminal domains of CI repressors are known to be cleaved by a bacterial ClpXP protease in *S. aureus*.^24^ This suggests the possibility of a non-canonical mechanism enabling truncated CIs to undergo SOS induction without self-cleavage.

**Fig. 4:**
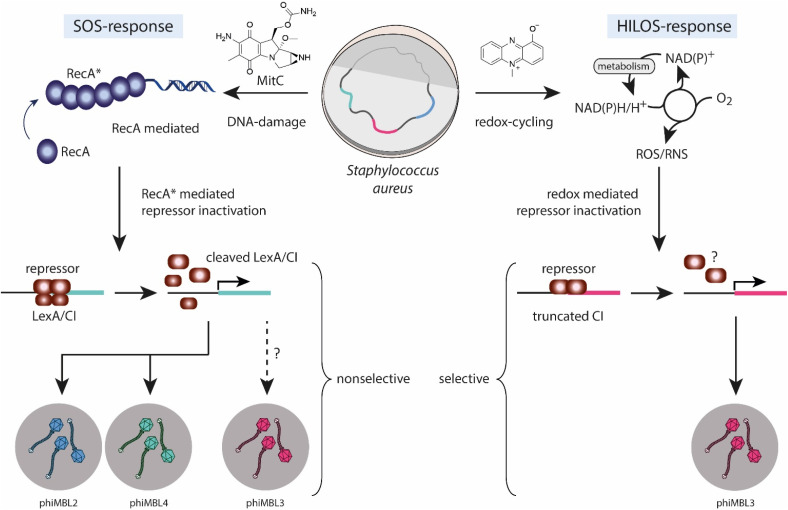
Mechanistic model of two distinct pathways for prophage induction in *S. aureus*: the generic SOS response and the phage-selective HiLOS response. The SOS response relies on DNA damage-induced RecA activation and cleavage of LexA/CI phage repressors, while the HiLOS response triggers prophage induction through an excessive ROS/RNS generation, involving a redox-mediated repressor inactivation.

## Conclusion

We report the discovery of three new potent selective prophage inducers (14–16). These phenazines were found to trigger selective induction in *S. aureus* ATCC 6341 in the same manner as pyocyanin involving redox cycling and considerable ROS generation. Furthermore, we have demonstrated that strong ROS formation initiates phiMBL3 production. We observed that ROS/RNS agents, such as hydroperoxides and a NONOate directly cause selective induction, suggesting an SOS-independent pathway we term the HiLOS response.

## Author contributions

M. J., T.-H. N. N. and T. B. conceived and designed the study. T.-H. N. N., C. R.-B. and F. A. synthesized the phenazines. M. J. performed the prophage induction, qPCR quantification, MIC assays, mutant generation, ROS detection and redox cycling experiments. S. J. did the anaerobic work, J. N. constructed the lysogen. A. W. and G. S. completed the CV measurements. All authors contributed to the interpretation and analysis of the data. M. J. and T. B. wrote the manuscript.

## Conflicts of interest

There are no conflicts to declare.

## Supplementary Material

SC-OLF-D5SC04923G-s001

## Data Availability

The data supporting this article have been included as part of the supplementary information (SI). Supplementary information: materials, experimental methods, syntheses, supporting tables and figures. See DOI: https://doi.org/10.1039/d5sc04923g.
