# Supplementary material for: A phage-selective trigger hints at an SOS-independent mechanism of prophage induction by oxidative stress
Source: Chem Sci. 2025 Nov 11;17(2):1151–7. doi: 10.1039/d5sc04923g (PMC12628705; doi:10.1039/d5sc04923g)
Supplement: SC-017-D5SC04923G-s001 [file SC-017-D5SC04923G-s001.pdf]

Supplementary Information (SI) for Chemical Science.
This journal is © The Royal Society of Chemistry 2025

Supporting Information

A phage-selective trigger hints at an SOS-independent mechanism of prophage induction by oxidative stress.

Magdalena Jancheva, Thi-Hong Nhung Nguyen, Felix Anderl, Shubham Joge, Jessica Neubauer, Clarissa Rominger-Baumann, Alexandra Walter, Golo Storch and Thomas Böttcher

1. Materials and methods

The solvents and chemicals for synthesis were purchased from Sigma-Aldrich, VWR Chemicals, Merck, TCI chemicals or ACROS ORGANICS and were used without purification. Pyocyanin and the phenazine compounds **14-18** were purchased from Merck. Thin-layer chromatography (TLC) was performed using aluminium sheets "TLC Silica gel 60 F_254" from Merck Millipore and analysed with UV light. For silica gel chromatography, distilled technical grades solvents, and silica gel 60 A (Carl Roth) was used. Automated flash chromatography was carried out on a Teledyne ISCO CombiFlash®Rf+ system with prepacked columns from RediSep®. For semi-preparative HPLC analysis, a LC-20AT Liquid Chromatograph of Shimadzu, equipped with a Reprosil 100 C18, 5 µM, 250 x 10 mm column (Dr. Maisch GmbH) was used. NMR spectra were obtained with Bruker Avance-III 400 and Bruker Avance-III 600 NMR spectrometers at ambient temperature. Multiplicities are given as follows: s-singlet, d-doublet, dd-doublet of doublets, ddd- doublet of doublets of doublets, t-triplet, tt- triplet of triplets, q-quartet, m-multiplet. Chemical shift (δ) is given in parts per million (ppm) relative to the solvent residues signal with $CDCl_3$-*d* (δ_H = 7.26 ppm and δ_C = 77.16 ppm), DMSO-*d*$_6$ (δ_H = 2.50 ppm and δ_C = 39.52 ppm) or MeOD-*d*$_4$ (δ_H = 3.31 ppm and δ_C = 49.00 ppm). The data obtained were processed and analysed with MestReNova 8.0 software. High resolution mass spectrometry (HR-MS) data were obtained on an ESI-Orbitrap (Thermo Scientific, LTQ Orbitrap Velos) by direct injection and analyzed with Xcalibur (Thermo Scientific) software.

2. Bacterial strains

Two strains were used in the study: *S. aureus* ATCC 6341 – a poly-lysogenic prophage host, and *S. aureus* RN4220 – an indication strain for plaque assays. Both were obtained from the German Collection of Microorganisms and Cell Cultures (DSMZ). Overnight cultures were prepared from glycerol cryostocks in LB medium (Roth) and incubated at 37 °C, 180 rpm. For the induction experiments, the prophage host was grown at the same temperature and shaking parameters in CCY medium (yeast

extract 30 g/L, casamino acids 20 g/L, sodium pyruvate 20 g/L, 2.5 g Na_2HPO_4, 0.42 g/L KH_2PO_4) unless otherwise indicated.

3. MIC determination, phage induction and plaque assays

From an overnight culture of *S. aureus* ATCC 6341, 1000-fold dilutions were made in CCY medium. To 99 µL of the diluted bacteria, 1 µL of each phenazine (from DMSO stock) was added in 96-well plates. After overnight incubation at 37 °C, 180 rpm the plates were visually accessed for bacterial growth. The lowest concentration at which no cell growth was observed, was considered as MIC. Three independent biological replicates were performed when determining the MIC of each phenazine.

For the induction assays, *S. aureus* ATCC 6341 was diluted 1:100 in CCY medium and grown to OD_{600} of 0.8. Each of the phenazines was then supplemented to portions of the culture at four different concentrations, in two-fold dilutions, starting with the MIC value or 200 µM. After 4 h incubation at 37 °C and 180 rpm, 1 mL was centrifuged and sterile filtered through 0.2 µm syringe filter to obtain the cell-free phage supernatant. These were stored at 4 °C until further use. For the anaerobic experiments, ATCC 6341 was grown and induced under same conditions in an anaerobic chamber (Coy Laboratory Products, USA) operated under 70% N_2, 25% CO_2, 5% H_2.

For the plaque assays, the indicator strain *S. aureus* RN4220 was grown overnight in LB medium. 300 µL of the undiluted overnight culture were mixed with 100 µL of the phenazine-induced phage supernatants in tubes containing 0.6% LB top agar. The top agar was then poured on 1.5% LB agar plates containing 10 mM $CaCl_2$. The plates were incubated at 37 °C and the next day plaque forming units (PFUs) were counted. Induction and quantification assays were performed in biological triplicates and the results are expressed as PFU/mL.

4. Pyocyanin mutants

Pyo^R mutants were generated as described previously,[1,2] by exposing *S. aureus* ATCC 6341 to various concentrations of pyocyanin (starting at 2 x MIC) for 14 consecutive days. Mutant DNA was extracted with the InvitrogenTM PureLinkTM Microbiome DNA purification kit (ThermoFisher Scientific) and genome re-sequencing analysis was performed at Microsynth AG (Balgach, Switzerland). Finally, the detected mutations were confirmed by Sanger sequencing at Eurofins GATC Biotech GmbH (Konstanz, Germany). Since all pyo^R mutants showed one common gene mutation, only one of them was used to examine the oxidoreductase-dependent mode of action of the newly identified phenazine prophage inducers (compounds **14-16**).

5. ROS detection, redox cycling and H_2O_2 quantification

2’,7’-dichlorofluorescein diacetate (DCF-DA) was utilized as a fluorogenic dye to detect ROS production upon treatment with the phenazines. *S. aureus* ATCC 6341 was grown to OD_{600} 0.8, cells were collected, washed with PBS and treated with 20 µM DCF-DA for 30 min at 37 °C. After a repeated washing step and re-suspension in a fresh medium, 99 µL were added to black Corning 96-well plates containing 1 µL of the phenazine compounds. Fluorescence (Ex/Em=485/535 nm) was measured on a Tecan microplate reader after 2 h plate incubation at 37 °C with shaking. All measurements were performed in triplicates and mean values with their standard deviations are reported.
The redox cycling ability of the phenazine compounds and mitomycin C was determined as previously described.[3] Briefly, 25 µM of each compound were mixed with 200 µM NADPH in phosphate buffer (pH 7). The absorbance spectra of the mixture in the range between 200-800 nm was measured on a Tecan Infinite 200 Pro instrument after 20 min or 6 h incubation. The absence of the peak at 340 nm indicated complete NADPH oxidation, hence a potent redox cycling ability.
To further confirm the results obtained by monitoring the absorbance spectra, quantification of H_2O_2 in the mixture (after different incubation times) was performed with the FOX (ferrous oxidation−xylenol orange) reagent. Accordingly, 180 µL of the reagent were added to 20 µL reaction mixture and after 30 min incubation at room temperature (RT) in the dark, absorbance at 586 nm was read out on a Tecan Infinite 200 Pro. The H_2O_2 concentration was determined by preparing a calibration curve in the range of 0.25-2.0 µM. Experiments were performed in three independent replicates and mean H_2O_2 concentrations with the corresponding standard deviations are given.

6. NAD^+/NADH quantification

S. aureus ATCC 6341 wild-type and the pyo^R mutant strain were grown to OD_{600} of 0.8 at LB medium, prior to treating them with 25 µM pyocyanin. After 10 min incubation at 37 ºC, the cells were pelleted by centrifugation, washed twice with PBS and lysed with FastPrep^{24TM} 5G (MP Biomedicals) at 6.0 m/s for 40 s. NAD^+ and NADH were extracted and quantified using the NAD/NADH assay kit by Merck (MAK468) according to the manufacturer instructions.

7. CV measurements

Electrochemical measurements were carried out with a Metrohm Autolab PGSTAT204 using glassy carbon (diameter = 3 mm) as a working electrode, a platinum wire as a counter electrode and $Ag/AgNO_3$ (0.01 M $AgNO_3$ in electrolyte) separated by a vycor frit as the reference electrode. A five-necked flask was used: One inlet was used to flush the cell with argon, one inlet was used for the addition of the samples, and the remaining inlets were fitted with the counter, working and reference electrodes. All

potentials are given vs. SCE. For referencing, ferrocene was used as an internal standard ($E_{1/2}(Fc^+/Fc)$ = 74 mV *vs.* $Ag/AgNO_3$)[4] and the potentials were adjusted to the SCE scale ($Ag/AgNO_3$ = 298 mV *vs.* SCE). All cyclic voltammograms (CVs) were measured with a scan rate of 0.5 V s^{-1}, unless otherwise noted. The measurements were carried out using pure acetonitrile (MeCN) as solvent and tetrabutylammonium hexafluorophosphate ($TBAPF_6$; c = 0.1 M) was used as the electrolyte.

8. qPCR – sample preparation and capsid gene detection

Sample preparation was conducted as previously reported with slight modifications.[5] Accordingly, 1 µL of the induced supernatants were diluted with 9 µL TMN buffer. To eliminate host DNA, 3 U of DNase I (Thermofisher Scientific) were subsequently added and the tubes were incubated at 37 °C for 1 h. Samples were then heated at 95 °C for 30 min to disassemble the capsid proteins and release the phage DNA. Finally, 100 µL of nuclease-free water was added and samples were stored at -20 °C overnight.
Next day, qPCR was performed targeting the capsid protein genes of phiMBL2-phiMBL4. The qPCR reaction wells contained: 3 µL dH_2O, 1 µL primers (Metabion AG, final concentration 250 nM), 1 µL sample and 5 µL SYBR green master mix (Thermofisher Scientific). The experiment was performed on a Roche 96 Lightcycler® instrument with the following setup: preincubation at 95°C (600 sec) → 2 step amplification 40·[95 °C (15 sec) → 60 °C (60 sec)] → high resolution melting [95 °C (60 sec) → 40 °C (60 sec) → 65 °C (1 sec) → 97 °C (1 sec)]. Obtained data was analysed on Lightcycler® 96 Software 1.1 (Roche).

9. RNLys(phiMBL3) generation

phiMBL3 lysogens were generated by a previously established protocol.[13] Briefly, phage supernatant induced by pyocyanin was spotted on a RN4220 lawn and the plate was incubated for few days at 30 ºC until colonies were formed within the plaques. Bacteria from these growth spots were then streaked onto fresh LB agar plates and incubated for 1-2 days until single colonies formed. The colonies were picked and grown in LB medium overnight, prior to DNA extraction and qPCR phage detection. The presence of phiMBL3 in RN4220 was additionally confirmed by genome sequencing at the Joint Microbiome Facility at the University of Vienna.

10. Statistical analysis

All statistical analyses were performed using GraphPad Prism (version 8, GraphPad Software, San Diego, CA, USA).

11. Syntheses

Synthesis of 3-methoxycyclohexa-3,5-diene-1,2-dione (22)

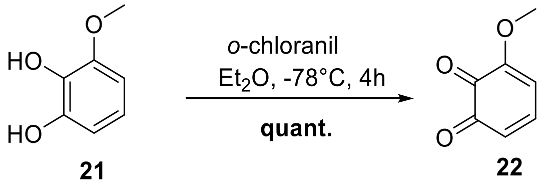

3-Methoxycatechol **21** (500 mg, 3.6 mmol, 1.0 eq.) was dissolved in diethyl ether (5 mL). The mixture was cooled to -78 °C. *o*-Chloranil (883 mg, 3.6 mmol, 1.0 eq.) was dissolved in diethyl ether (5 mL) and added dropwise to the reaction mixture. The solution was stirred for 4 h at -78 °C. The reaction mixture was filtered giving the desired benzoquinone **22** as a brown solid in quantitative yield, which was used without further purification.

General synthesis of 1-methoxyphenazines (1,6,7)

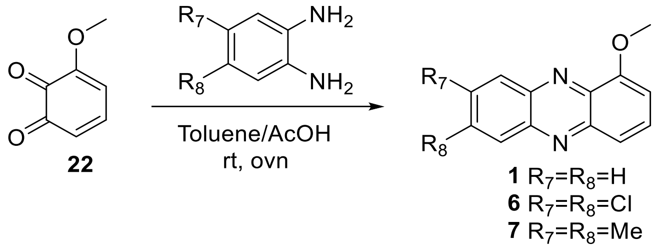

o-Phenylenediamine and derivatives (1.0 eq.), toluene and glacial acetic acid (v/v 1:1, 0.15 M) were added to the flask containing benzoquinone **22**. The mixture was stirred for 24 h at room temperature. Upon completion, the reaction mixture was neutralized with saturated sodium bicarbonate solution, washed with brine and extracted with DCM. The organic layers were dried with sodium sulfate, filtered and concentrated *in vacuo*. The crude products were purified via column chromatography with silica gel and DCM to yield desired derivatives.

1-Methoxyphenazine **1**: yellow brown solid (17% yield).

TLC*:* *Rf* = 0.4 (Hexane/EtOAc 1:2)
^{1}H NMR (400 MHz, $CDCl_3$-*d*) δ 8.40 (m, 1H), 8.24 (m, 1H), 7.88 – 7.80 (m, 3H), 7.75 (t, *J* = 8.9, 7.5 Hz, 1H), 7.08 (d, *J* = 7.5, 1.1 Hz, 1H), 4.18 (s, 3H).
^{13}C NMR (101 MHz, $CDCl_3$-*d*) δ 155.3, 144.2, 143.4, 142.4, 137.1, 131.1, 130.8, 130.4, 130.4, 129.3, 121.4, 106.6, 56.6.
HRMS: calc. for $C_{13}H_{10}N_2O$ +H^+ $[M+H]^+$ 211.0866, found: 211.0866

7,8-Dichloro-1-methoxyphenazine **6**: yellow solid (15% yield).

TLC*: Rf* = 0.4 (DCM)
^{1}H NMR (400 MHz, $CDCl_3$-*d*) δ 8.54 (s, 1H), 8.38 (s, 1H), 7.84 – 7.74 (m, 2H), 7.10 (dd, *J* = 6.2, 2.5 Hz, 1H), 4.18 (s, 3H)
^{13}C NMR (101 MHz, $CDCl_3$-*d*) δ 155.2, 144.3, 141.7, 140.6, 137.3, 136.0, 135.2, 131.7, 130.3, 129.4, 121.2, 107.3, 56.6.
HRMS: calc. for $C_{13}H_8Cl_2N_2O$ +H^+ $[M+H]^+$: 279.0086, found: 279.0089

7,8-Dimethyl-1-methoxyphenazine **7**: yellow solid (20% yield).

TLC*: Rf* = 0.2 (DCM/MeOH 200:1)
^{1}H NMR (400 MHz, $CDCl_3$-*d*) δ 8.14 (s, 1H), 7.97 (s, 1H), 7.81 (d, *J* = 8.8 Hz, 1H), 7.69 (t, *J* = 7.6 Hz, 1H), 7.03 (d, *J* = 7.6 Hz, 1H), 4.16 (s, 3H), 2.55 (s, 6H).
^{13}C NMR (101 MHz, $CDCl_3$-*d*) δ 155.2, 143.7, 142.9, 142.5, 141.8, 141.6, 136.5, 129.9, 128.7, 127.6, 121.3, 106.1, 56.5, 20.8, 20.8.
HRMS: calc. for $C_{15}H_{14}N_2O$ +H^+ $[M+H]^+$: 239.1179, found: 239.1177

Synthesis of 4-bromo-1-methoxyphenazine (2)

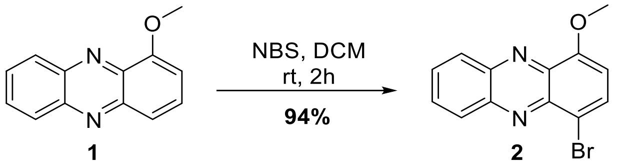

1-Methoxyphenazine **1** (50 mg, 0.24 mmol, 1.0 eq.) was dissolved in dichloromethane (15 mL), before *N*-bromosuccinimide (85 mg, 0.48 mmol, 2.0 eq.) was added. The reaction was allowed to stir at room temperature for 2 h. Upon completion, the reaction mixture was diluted with DCM, washed with brine and extracted with DCM. The combined organic layers were dried with sodium sulfate, filtered and concentrated *in vacuo*. Purification was performed via column chromatography using silica gel and DCM. Further purification was conducted with automated flash chromatography using a PE:EtOAc gradient (0-100% EtOAc) to afford 65 mg of 4-bromo-1-methoxyphenazine **2** as a yellow, slightly orange solid (94% yield).

TLC*: Rf* = 0.34 (DCM)
^{1}H NMR (400 MHz, $CDCl_3$-*d*) δ 8.48 – 8.42 (m, 1H), 8.42 – 8.36 (m, 1H), 8.09 (d, *J* = 8.3 Hz, 1H), 7.94 – 7.85 (m, 2H), 6.97 (d, *J* = 8.2 Hz, 1H), 4.18 (s, 3H).
^{13}C NMR (101 MHz, $CDCl_3$-*d*) δ 155.3, 143.8, 142.4, 141.2, 137.3, 133.3, 131.5, 131.2, 130.0, 130.0, 114.4, 107.0, 56.8.
HRMS: calc. for $C_{13}H_9BrN_2O$ +H^+ $[M+H]^+$: 288.9971, found: 288.9972

Synthesis of 4-chloro-1-methoxyphenazine (3) and 2,4-dichloro-1-methoxyphenazine (4)

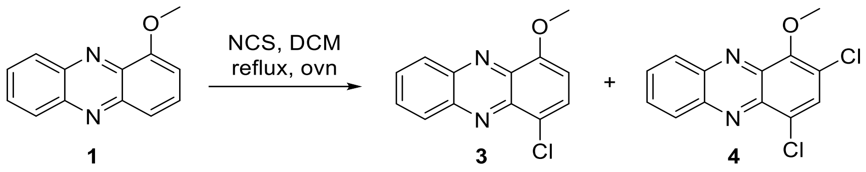

1-Methoxyphenazine **1** (50 mg, 0.24 mmol, 1.0 eq.) was dissolved in DCM (5 mL) before *N*-chlorosuccinimide (70.8 mg, 0.53 mmol, 2.2 eq.) was added. The reaction was stirred under reflux overnight. Upon completion, the reaction mixture was diluted with DCM, washed with brine and extracted with DCM. The combined organic layers were dried with sodium sulfate, filtered and concentrated *in vacuo*. Purification was performed via column chromatography using silica gel and DCM:EtOAC 7:1. Further purification using automated flash chromatography using PE:EtOAc (0-100% EtOAc) was performed to obtain 4-chloro-1-methoxyphenazine **3** as a yellow solid (31% yield), and 2,4-dichloro-1-methoxyphenazine **4** as a light yellow solid (28% yield), respectively.

4-Chloro-1-methoxyphenazine **(3)**

TLC*: Rf* = 0.7 (DCM/EtOAc 7:1)
^{1}H NMR (400 MHz, $CDCl_3$-*d*) δ 8.43 – 8.34 (m, 2H), 7.94 – 7.81 (m, 3H), 6.99 (d, *J* = 8.3 Hz, 1H), 4.17 (s, 3H).
^{13}C NMR (101 MHz, $CDCl_3$-*d*) δ 154.4, 143.3, 142.3, 140.4, 137.1, 131.4, 131.1, 130.0, 129.8, 129.6, 124.0, 106.1, 56.6.
HRMS: calc. for $C_{13}H_9ClN_2O$ +H^+ $[M+H]^+$: 245.0476, found: 245.0476

2,4-Dichloro-1-methoxyphenazine **(4)**

TLC*: Rf* = 0.9 (DCM/EtOAc 7:1)
^{1}H NMR (400 MHz, $CDCl_3$-*d*) δ 8.39 – 8.30 (m, 2H), 7.95 (s, 1H), 7.94 – 7.88 (m, 2H), 4.31 (s, 3H).
^{13}C NMR (101 MHz, $CDCl_3$-*d*) δ 151.0, 143.1, 143.0, 139.7, 139.3, 132.0, 131.7, 131.1, 130.1, 130.0, 128.7, 126.2, 62.9.
HRMS: calc. for $C_{13}H_8Cl_2N_2O$ +H^+ $[M+H]^+$:279.0086, found: 279.0087

Synthesis of 4-iodo-1-methoxyphenazine (5)

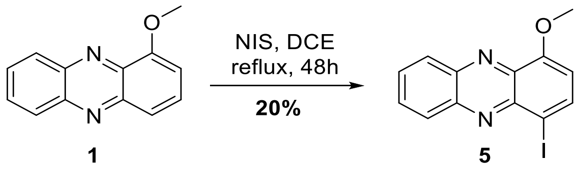

1-Methoxyphenazine **1** (50 mg, 0.24 mol, 1.0 eq.) was dissolved in 1,2-dichloroethane (10 mL) before *N*-iodosuccinimide (107 mg, 0.48 mol, 2.0 eq.) was added. The reaction was brought to reflux and was allowed to stir for 48 hours. Upon completion, the reaction mixture was washed with brine and extracted with DCM. The combined organic layers were dried with sodium sulfate, filtered and concentrated *in vacuo*. Purification was conducted with column chromatography using silica gel and DCM to afford 19 mg of 4-iodo-1-methoxyphenazine **5** as a yellow solid (20% yield).

TLC: *Rf* = 0.6 (DCM)
^{1}H NMR (400 MHz, $CDCl_3$-*d*) δ 8.48 – 8.42 (m, 1H), 8.38 (dd, *J* = 7.6, 2.7 Hz, 2H), 7.94 – 7.85 (m, 2H), 6.90 (d, *J* = 8.2 Hz, 1H), 4.18 (s, 3H)
^{13}C NMR (101 MHz, $CDCl_3$-*d*) δ 156.2, 144.0, 142.6, 142.5, 140.1, 136.9, 131.3, 131.0, 129.7, 108.1, 90.9, 56.7. Due to overlap one signal concealed.
HRMS: calc. for $C_{13}H_9IN_2O$ +H^+ $[M+H]^+$:336.9832, found: 336.9828

Synthesis of 1-hydroxyphenazine (8)

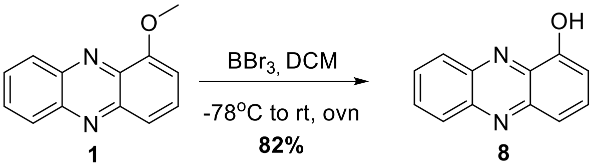

1-Methoxyphenazine **1** (156 mg, 0.74 mmol, 1.0 eq.) was dissolved in anhydrous DCM. The mixture was cooled to -78 °C, before the dropwise addition of boron tribromide (3.9 mL, 3.9 mmol, 5.2 eq., 1.0 M in DCM). The reaction mixture was left to stir at -78 °C for 1 h, before it was allowed to reach ambient temperature overnight. Upon completion, the solution was transferred to a separation funnel containing saturated sodium bicarbonate solution and extracted with DCM. The combined organic layers were dried over sodium sulfate, filtered and concentrated *in vacuo*. The resulting solid was purified via column chromatography using silica gel and DCM to obtain 197 mg of 1-hydroxyphenazine **8** as a yellow solid (82% yield).

TLC: *Rf* = 0.25 (DCM)
^{1}H NMR (400 MHz, MeOD-d_4) δ 8.34 – 8.26 (m, 1H), 8.21 – 8.12 (m, 1H), 7.94 – 7.84 (m, 2H), 7.77 (dd, *J* = 8.9, 7.4 Hz, 1H), 7.68 (dd, *J* = 8.8, 1.2 Hz, 1H), 7.18 (dd, *J* = 7.4, 1.2 Hz, 1H).
^{13}C NMR (101 MHz, MeOD- d_4) δ 154.5, 145.0, 144.5, 143.0, 137.1, 133.3, 132.4, 131.5, 130.6, 129.8, 120.0, 111.1.
HRMS: calc. for $C_{12}H_8N_2O$ +H^+ $[M+H]^+$ 197.0709, found: 197.0708

Synthesis of 4-bromophenazin-1-ol (10)

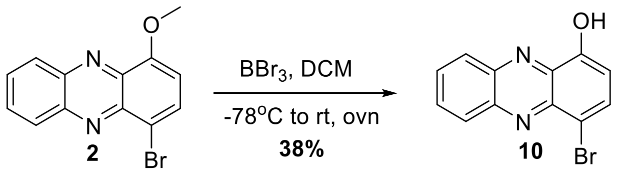

4-Bromo-1-methoxyphenazine **2** (30 mg, 0.10 mmol, 1.0 eq.) was dissolved in anhydrous DCM (2.4 mL). The mixture was cooled to -78 °C before the dropwise addition of boron tribromide (0.31 mL, 0.31 mmol, 3.0 eq., 1.0 M in DCM). The reaction mixture was stirred at -78 °C for 1 h before it was allowed to warm up to room temperature overnight. Upon completion, the reaction solution was transferred to a separation funnel containing saturated sodium bicarbonate solution and extracted with DCM. The combined organic layers were dried with sodium sulfate, filtered, and concentrated *in vacuo*. The crude residue was purified via column chromatography with silica gel and hexane/EtOAc 3:1. Further purification was performed via automated flash chromatography using PE:EtOAc gradient (0-100% EtOAc) to obtain 11 mg of 4-bromophenazin-1-ol **10** as a yellow solid (38% yield).

TLC*: Rf* = 0.18 (Hexane/EtOAc 3:1)
^{1}H NMR (400 MHz, $CDCl_3$-*d*) δ 8.46 – 8.37 (m, 1H), 8.29 – 8.23 (m, 1H), 8.22 (s, 1H), 8.09 (d, *J* = 8.1 Hz, 1H), 7.97 – 7.84 (m, 2H), 7.14 (d, *J* = 8.1 Hz, 1H).
^{13}C NMR (101 MHz, $CDCl_3$-*d*) δ 151.8, 144.5, 141.4, 141.0, 135.1, 134.6, 131.6, 131.5, 130.3, 129.0, 112.3, 109.5.
HRMS: calc. for $C_{12}H_7BrN_2O$ +H^+ $[M+H]^+$:274.9815, found: 274.9817

Synthesis of 2,4-dibromophenazin-1-ol (9)

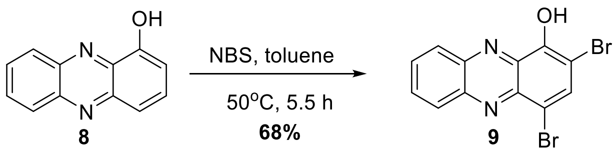

1-Hydroxyphenazine **8** (150 mg, 0.76 mmol, 1.0 eq.) was dissolved in toluene (17.6 mL) and *N*-bromosuccinimide (300 mg, 1.67 mmol, 2.2 eq.) was added. The mixture was heated to 50 °C for 5.5 h. After completion, the reaction was allowed to cool to room temperature and concentrated *in vacuo.* Column chromatography using silica gel and DCM was conducted. Further purification was performed on a NP-HPLC with hexane/EtOAc gradient (0-100% EtOAc, flow rate 15 mL/min) to obtain 191 mg of 2,4-dibromophenazin-1-ol **9** as a yellow solid (68% yield).

TLC*: Rf* = 0.15 (DCM)
^{1}H NMR (400 MHz, $CDCl_3$-*d*) δ 8.53 (br s, 1H), 8.44 – 8.38 (m, 1H), 8.30 – 8.26 (m, 1H), 8.26 (s, 1H), 7.99 – 7.88 (m, 2H).
^{13}C NMR (101 MHz, $CDCl_3$-*d*) δ 149.2, 144.3, 141.6, 140.1, 137.3, 134.4, 132.3, 131.8, 130.4, 128.9, 113.1, 103.2.
HRMS: calc. for $C_{12}H_6Br_2N_2O$ +H^+ $[M+H]^+$ 354.8899, found: 354.8899

General synthesis of 2,4-dibromophenazin-1-ol esters (11, 12, 13)

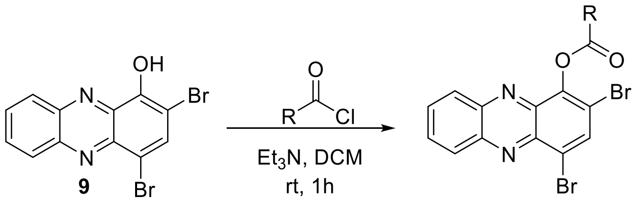

11 R=Methyl
12 R=Phenyl
13 R=Cyclohexyl

2,4-Dibromophenazin-1-ol **9** (1.0 eq.) was dissolved in DCM (0.03 M). Triethylamine (4.9 eq.) was added, followed by the addition of the respective acid chloride (3.4 eq.) at room temperature. The solution was allowed to stir for 1 h, and was then quenched with saturated sodium bicarbonate solution. The resulting mixture was extracted with DCM. The organic layers were dried with sodium sulfate, filtered and concentrated *in vacuo*. Purification via column chromatography using silica gel and hexane/EtOAc 9:1 afforded the desired product. Further purification was performed using NP-HPLC with hexane/EtOAc gradient (0-100% EtOAc, flow rate 15mL/min) if needed.

2,4-Dibromophenazin-1-ol acetate **11**: yellow solid (54% yield).

TLC*: Rf* = 0.18 (Hexane/ EtOAc 9:1)
^{1}H NMR (400 MHz, $CDCl_3$-*d*) δ 8.39 – 8.33 (m, 2H), 8.29 – 8.22 (m, 1H), 7.96 – 7.87 (m, 2H), 2.62 (s, 3H).
^{13}C NMR (101 MHz, $CDCl_3$-*d*) δ 168.3, 145.2, 143.7, 143.5, 140.3, 137.8, 135.8, 132.3, 132.0, 130.2, 129.9, 122.3, 117.1, 20.8.
HRMS: calc. for $C_{14}H_8Br_2N_2O_2$ +H^+ $[M+H]^+$:396.9005, found: 396.9009

2,4-Dibromophenazin-1-ol benzoate **12**: yellow solid (78% yield).

TLC*: Rf* = 0.1 (Hexane/EtOAc 19:1)
^{1}H NMR (400 MHz, $CDCl_3$-*d*) δ 8.43 – 8.33 (m, 4H), 8.17 – 8.13 (m, 1H), 7.91 (ddd, *J* = 8.7, 6.6, 1.5 Hz, 1H), 7.84 (ddd, *J* = 8.2, 6.6, 1.5 Hz, 1H), 7.77 – 7.71 (m, 1H), 7.62 (dd, *J* = 8.4, 7.1 Hz, 2H).

^{13}C NMR (101 MHz, $CDCl_3$-*d*) δ 164.2, 145.5, 143.7, 143.6, 140.3, 138.0, 135.8, 134.2, 132.1, 132.0, 130.9, 130.1, 128.9, 128.9, 122.3, 117.3, 103.2.
HRMS: calc. for $C_{19}H_{10}Br_2N_2O_2$ +H^+ $[M+H]^+$: 458.9161, found: 458.9164

2,4-Dibromophenazin-1-ol cyclohexanecarboxylate **13**: yellow solid (41% yield).

TLC*: Rf* = 0.15 (Hexane/EtOAc 19:1)
^{1}H NMR (400 MHz, $CDCl_3$-*d*) δ 8.40 – 8.30 (m, 2H), 8.25 – 8.16 (m, 1H), 7.96 – 7.84 (m, 2H), 2.94 (tt, 1H), 2.32 (dd, *J* = 13.1, 4.0 Hz, 2H), 2.02 – 1.68 (m, 5H), 1.53 – 1.33 (m, 3H).
^{13}C NMR (101 MHz, $CDCl_3$-*d*) δ 173.3, 145.3, 143.6, 143.5, 140.3, 137.8, 135.8, 132.1, 131.9, 130.1, 129.9, 121.9, 117.0, 43.3, 29.4, 26.0, 25.5.
HRMS: calc. for $C_{19}H_{16}Br_2N_2O_2$ +H^+ $[M+H]^+$ 464.9636, found: 464.9632

Synthesis of 4-bromo-1-methoxy-5-methylphenazin-5-ium methanesulfonate (19)

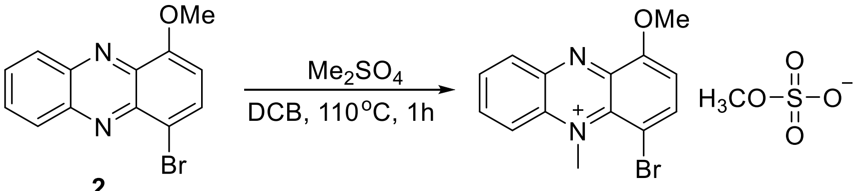

2
19

A suspension of 1-bromo-4-methoxyphenazine **2** (1.45 g, 5 mmol, 1.0 eq.) in 1,2-dichlorobenzene (5 mL, 1.1 M) was prepared and heated to 140 °C to maintain solubility, before cooling down to 110 °C. A solution of dimethyl sulfate (0.52 mL, 5.5 mmol, 1.1 eq.) in 1,2-dichlorobenzene (1.1 mL, 5.5 M) was added dropwise to the reaction mixture at 110 °C. The mixture was stirred for 1 h at 110 °C. Upon completion, the reaction mixture was cooled quickly in an ice bath and kept in a fridge for 3 days. The precipitated phenazinium methosulfate **19** was filtered off, washed with diethyl ether, and then dried *in vacuo* to yield the product (200 mg, 10% yield).

^{1}H NMR (400 MHz, DMSO-d_6) δ 8.31 (ddd, *J* = 12.0, 8.0, 2.8 Hz, 2H), 8.24 (d, *J* = 8.3 Hz, 1H), 8.08 – 7.96 (m, 2H), 7.21 (d, *J* = 8.4 Hz, 1H), 6.78 (s, 3H), 4.08 (s, 3H), 2.56 (s, 3H).
^{13}C NMR (101 MHz, DMSO-d_6) δ 155.0, 142.6, 141.7, 140.1, 136.7, 134.0, 132.1, 131.5, 129.5, 129.1, 112.4, 108.0, 56.3, 52.8, 29.5.
HRMS: calc. for $C_{14}H_{12}BrN_2O$ +H^+ $[M+H]^+$ 303.0133, found: 303.0126

Synthesis of 9,10-dimethylacridin-10-ium methanesulfonate (20)

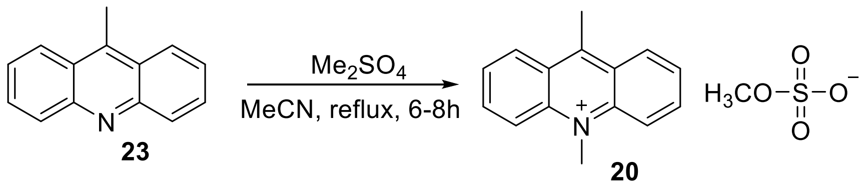

9-Methylacridine **23** (200 mg, 1.03 mmol, 1.0 eq.) was added to acetonitrile (10.3 mL, 0.1 M). Dimethyl sulfate (108 µL, 1.14 mmol, 1.1 eq.) was added at room temperature. The reaction mixture was stirred at reflux for around 6-8 h. Upon completion, the reaction mixture was cooled to room temperature and evaporated to dryness. The remaining crude solid was recrystallized from MeCN/MeOH 4:1 to yield methylacridinium methosulfate **20** as dark green solid (316 mg, 96% yield).

^{1}H NMR (400 MHz, DMSO-d_6) δ 8.92 (d, J = 7.9 Hz, 2H), 8.76 (d, J = 9.0 Hz, 2H), 8.43 (t, J = 7.8 Hz, 2H), 8.03 (t, J = 6.5 Hz, 2H), 4.82 (s, 3H), 3.53 (s, 3H), 3.37 (s, 3H).
^{13}C NMR (101 MHz, DMSO- d_6) δ 150.7, 141.3, 139.0, 131.6, 127.7, 126.3, 118.9, 52.8, 38.5, 16.5.
HRMS: calc. for $C_{15}H_{14}N$ +H^+ $[M+H]^+$ 208.1126, found: 208.1119

12. Supporting tables and figures

Table S1. Concentrations of hydrogen peroxide (H_2O_2) detected by the FOX reagent. Experiments were performed in three independent replicates and mean H_2O_2 concentrations with the corresponding standard deviations are given.

Compound	Concentration	Incubation time
**Pyo**	3.48 µM ± 0.73 µM	20 min
**MitC**	n.d.	6 h
**1**	n.d.	6 h
**2**	n.d.	6 h
**3**	n.d.	6 h
**4**	n.d.	6 h
**5**	n.d.	6 h
**6**	n.d.	6 h
**7**	n.d.	6 h
**8**	n.d.	6 h
**9**	n.d.	6 h
**10**	n.d.	6 h
**11**	n.d.	6 h
**12**	n.d.	6 h
**13**	n.d.	6 h
**14**	7.39 µM ± 1.76 µM	20 min
**15**	7.52 µM ± 1.67 µM	20 min
**16**	7.73 µM ± 1.47 µM	20 min
**17**	n.d.	6 h
**18**	n.d.	6 h
**19**	n.d.	20 min
	0.66 µM ± 0.15 µM	60 min
	1.18 µM ± 0.27 µM	90 min
	1.54 µM ± 0.35 µM	120 min
	1.89 µM ± 0.52 µM	180 min
	1.88 µM ± 0.56 µM	6 h
**20**	n.d.	6 h

n.d. = not detected

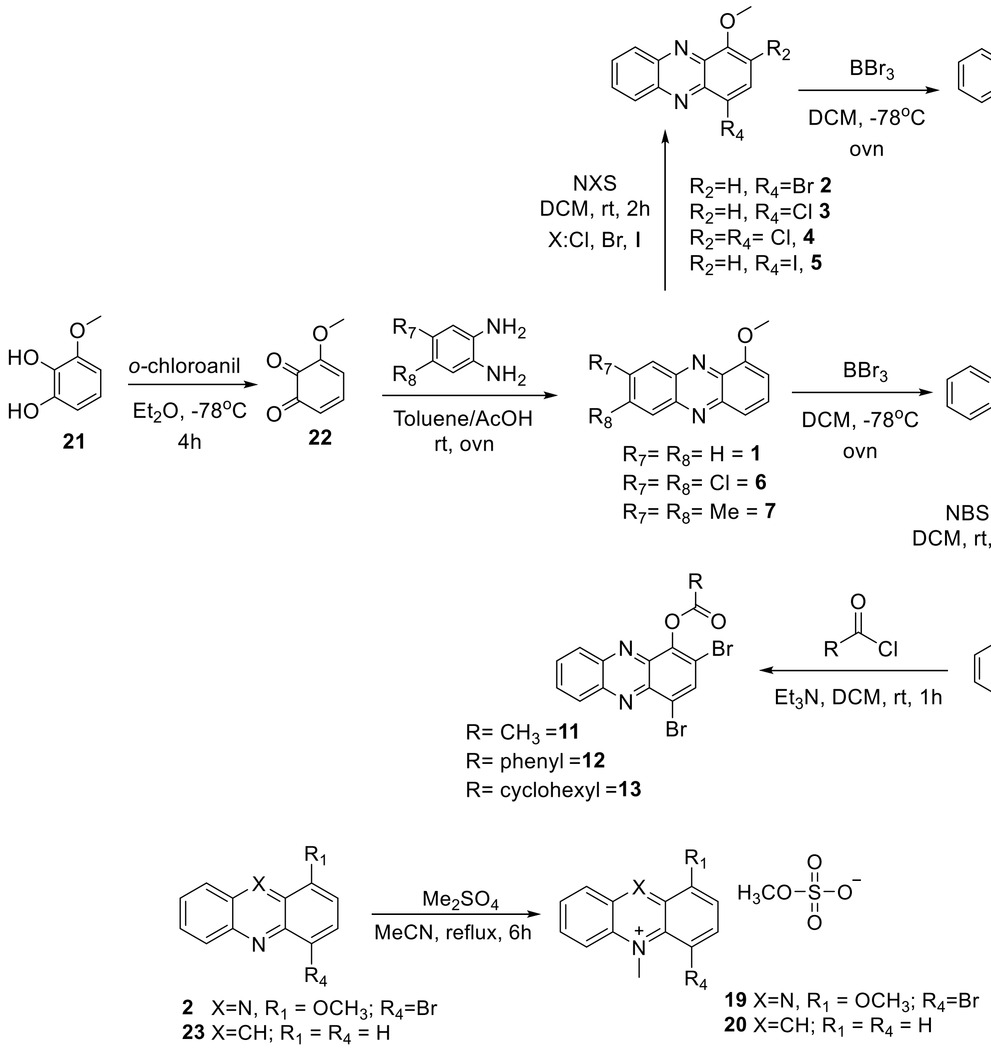

Figure S1. Scheme for the phenazine synthesis. The synthetic routes utilized to obtain the desired compounds is partially adapted from previous work by Garrison et.al.[6]

Compound	MIC (µM)	Compound	MIC (µM)
Pyo	50	11	3
1	>200	12	3
2	>200	13	>200
3	>200	14	50
4	>200	15	100
5	>200	16	200
6	>200	17	>200
7	>200	18	>200
8	>200	19	>200
9	3	20	>200
10	50		

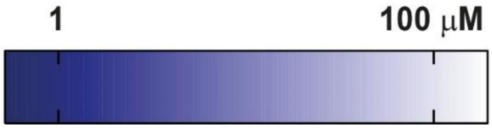

Figure S2. Heatmap representing the MIC values for pyocyanin and the phenazine derivatives **1-20** against *S. aureus* ATCC 6341.

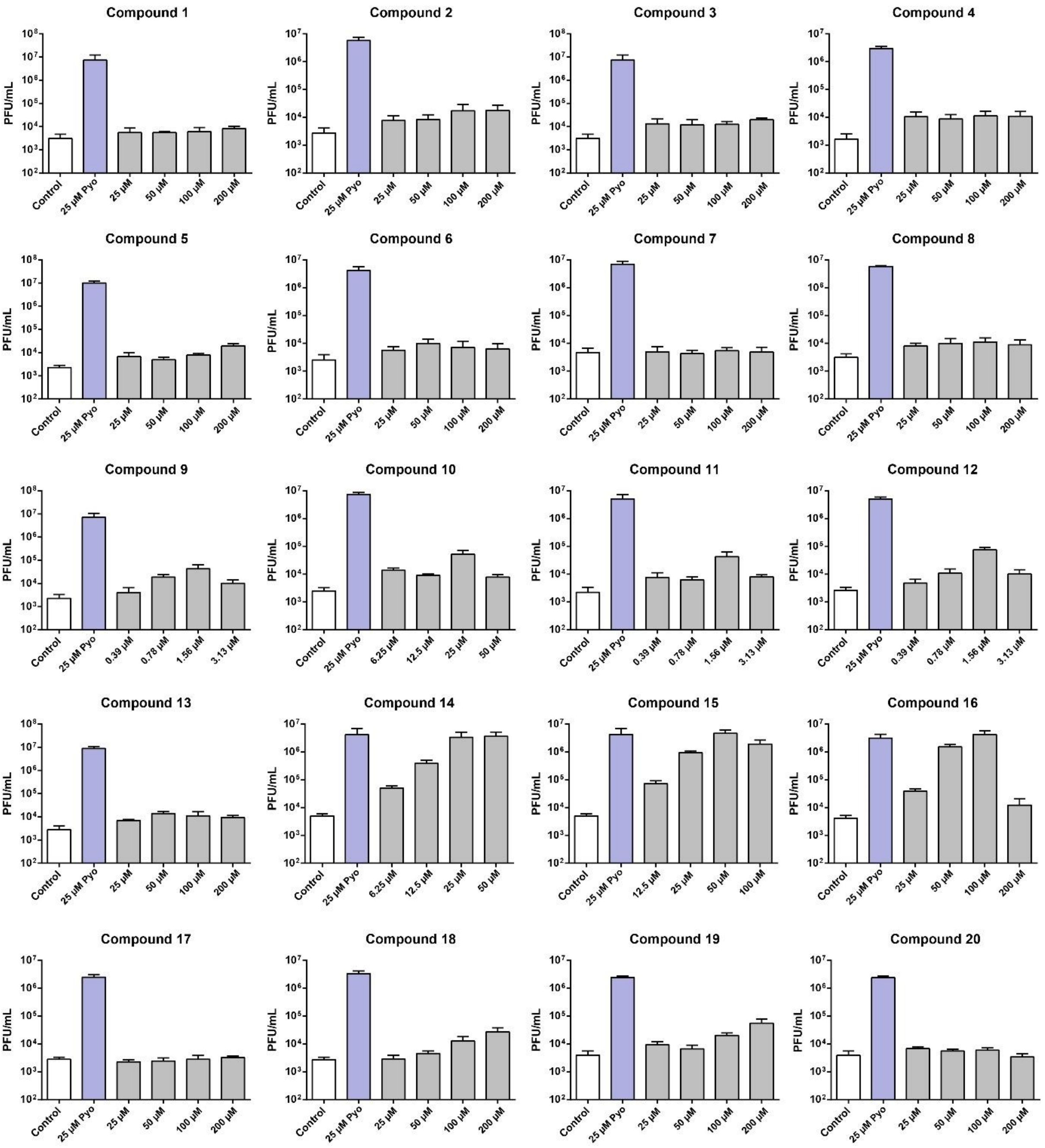

Figure S3. Phage titre counts for phenazine-treated supernatants in *S. aureus* ATCC 6341. The bacterial strain was treated with four different compound concentrations starting with 200 µM or the corresponding MIC value. Three other concentrations, which correspond to 1/2, 1/4 and 1/8 of the highest selected ones, were also tested in the assays. Induction experiments were performed in triplicates and the mean values with the corresponding standard deviations are presented.

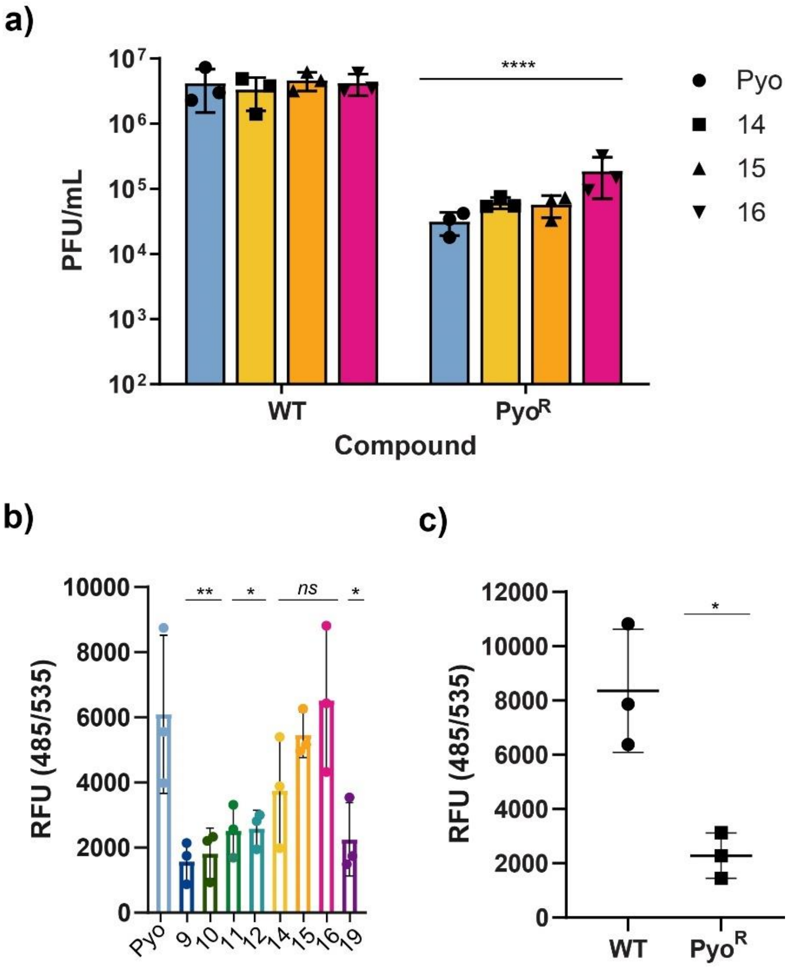

Figure S4. a) PFU/mL values for the *S. aureus* ATCC 6341 wild type and oxidoreductase-deficient mutant pyo^{R} upon induction with pyocyanin and compounds **14-16**. Two-way ANOVA was applied for the statistical analysis, followed by a Tukey's test. ****, P<0.0001 vs. WT. **b)** Quantification of ROS for the potent prophage inducers and the halogenated phenazines at the highest inducing concentrations. The results are expressed as relative fluorescence units (RFU) after subtraction of the RFU value for the non-treated control. Statistical significance was determined by one-way ANOVA, followed by Dunnett's comparison test. **, P<0.01; *, P<0.05; *ns*, not significant vs. pyocyanin. **c)** RFU measurements for the wild-type and pyo^{R} mutant strain in the presence of 25 µM pyocyanin. Statistical significance was determined using an unpaired two-tailed Student's t-test. *, P<0.05 compared with WT pyocyanin. **a), b) and c)** Three independent biological replicates were performed for each, and the mean values with the corresponding standard deviations are given.

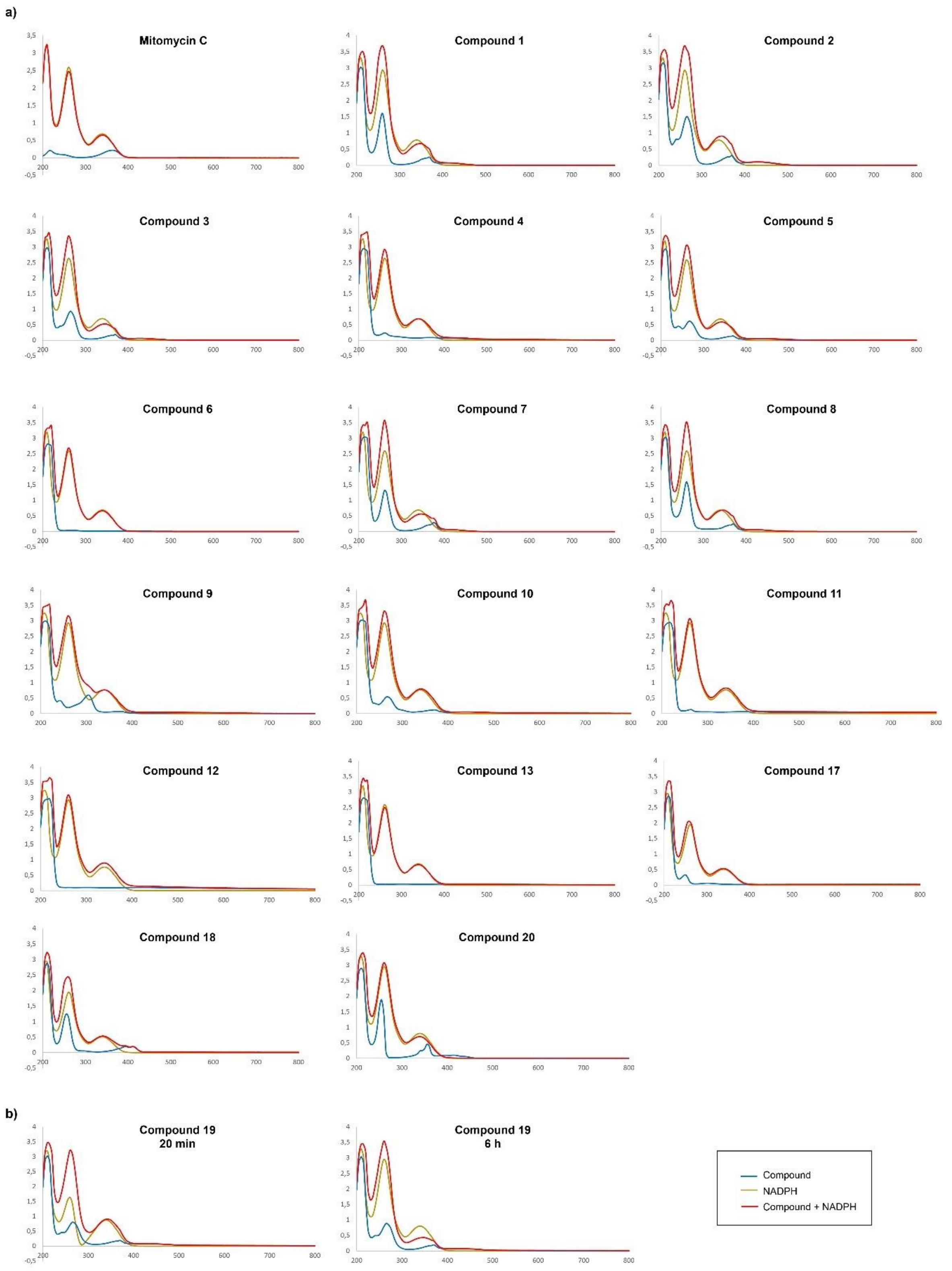

Figure S5. Absorption spectra of experiments with NADPH incubated with mitomycin C or with the weak prophage inducing phenazines **1-13** and **17-20**. **a)** and **b)** Blue lines represent the absorption of the distinctive compounds and yellow lines the absorption of NADPH alone. Red lines indicate the absorption spectra of the reducing agent NADPH in the presence of the phenazine derivatives.

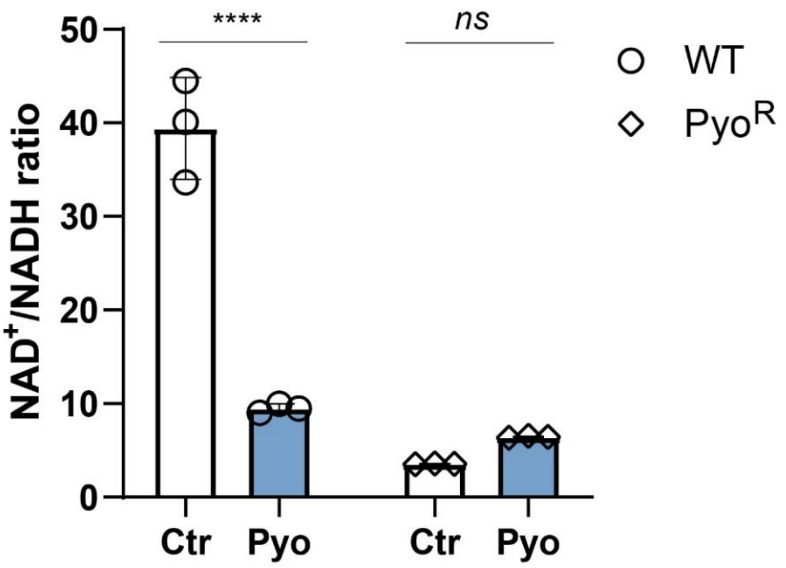

Figure S6. NAD^+/NADH ratios of wild-type *S. aureus* ATCC 6341 and its pyo^R mutant strain. Error bars represent standard deviations of three biological replicates. Statistical analysis was performed using two-way ANOVA, followed by a Tukey's test. ****, P<0.0001; *ns,* not significant.

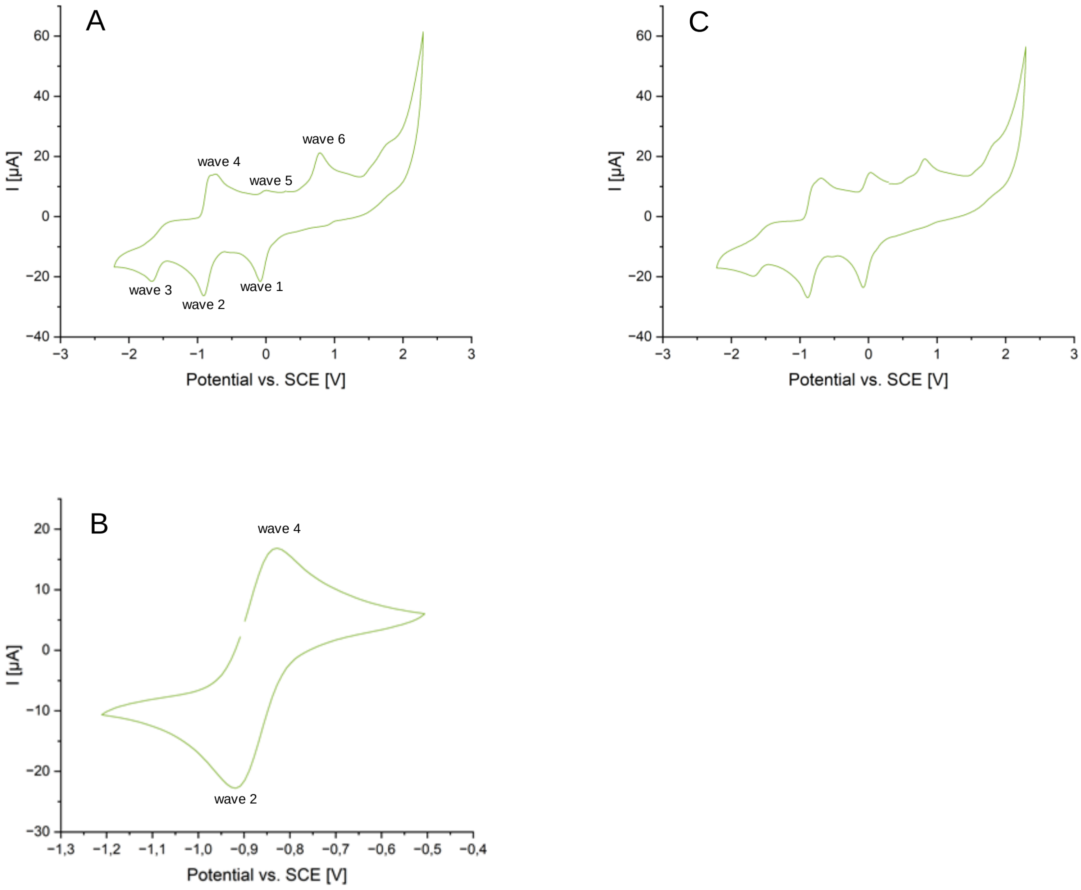

Figure S7. Cyclic voltammogram of pyocyanin (Pyo). **A)** In accordance with literature,[7] the reduction waves 2 and 3 were assigned to the stepwise reduction of neutral pyocyanin (Pyo) to $Pyo^{\bullet-}$ and Pyo^{2-}, respectively. For the reduction to the anionic radical $Pyo^{\bullet-}$, a reversible re-oxidation was observed **(B)** with a half-wave potential $E_{1/2}(Pyo/Pyo^{\bullet-})$ = –0.88 V vs. SCE. This value approximately matches the reported value (–0.78 V vs. SCE). We noted that the shape of the voltammograms changed after several CV scans, and the peaks broadened **(C)**. This points towards analyte decomposition and deposition on the surface of the electrodes.[8,9] A partly irreversible reduction was observed for the transformation of $Pyo^{\bullet-}$ to Pyo^{2-} at E_P = –1.66 V vs. SCE, which is in accordance with the reported value of –1.64 V vs. SCE. The irreversibility can be explained by a fast chemical reaction following the reduction, which presumably is the fast protonation of Pyo^{2-} to $PyoH^{-}$ in presence of a proton source such as residual water in the solvent.

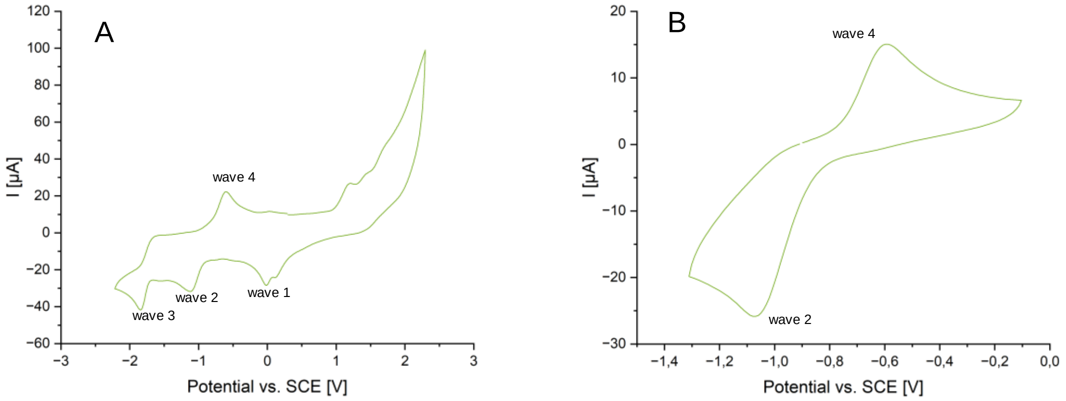

Figure S8. Cyclic voltammogram of compound **8** (1-hydroxyphenazine). **A**) A cyclic voltammogram was recorded at a scan rate of 0.5 V s^{-1}. **B**) Based on the detailed study by Sawyer,[7] we assign wave 2 to an irreversible reduction of 1-hydroxyphenazine (**8**) at a peak potential of E_P = –1.10 V *vs.* SCE (reported value: –0.92 V *vs.* SCE).

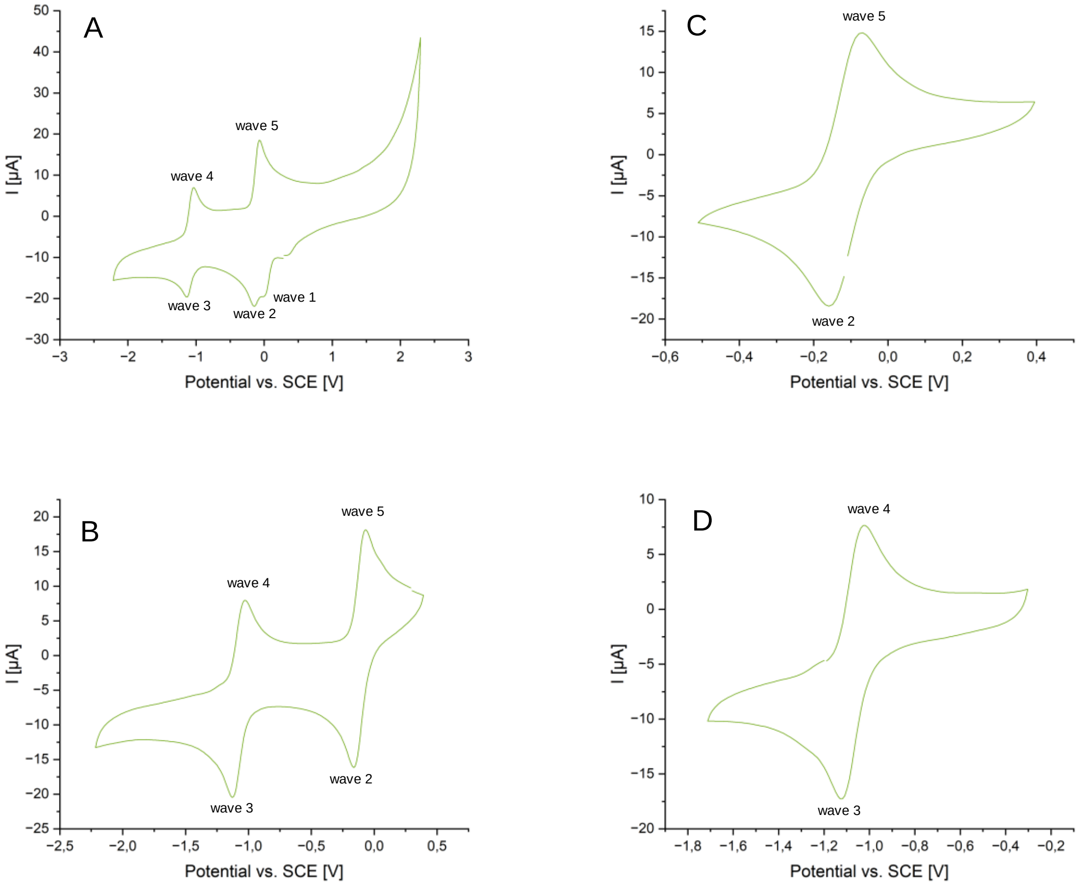

Figure S9. Cyclic voltammogram of compound **14•**OCH$_3$SO$_3$. **A)** Two reversible reductions are observed, which can be assigned to the one electron reduction to the radical $\mathbf{14}^{\bullet}$ with $E_{1/2}$ ($\mathbf{14}^{+}/\mathbf{14}^{\bullet}$) = –0.09 V *vs.* SCE **(B)**, and the one-electron reduction of radical $\mathbf{14}^{\bullet}$ to $\mathbf{14}^{-}$ with $E_{1/2}$ ($\mathbf{14}^{\bullet}/\mathbf{14}^{-}$) = –1.07 V *vs.* SCE **(C)**. Both redox potentials are in accordance with reported values for related *N*-methylphenazinium cations (–0.05 V and –1.02 V *vs.* SCE).[7]

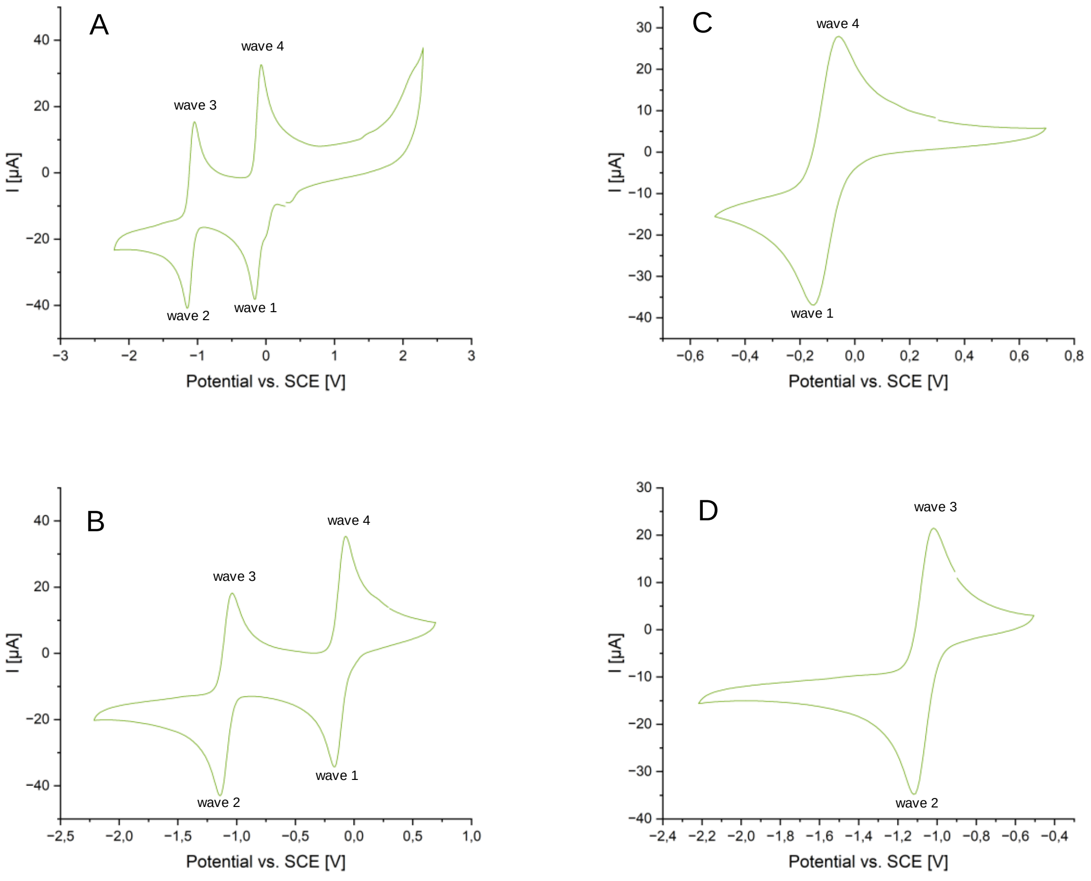

Figure S10. Cyclic voltammogram of compound **15**•$OCH_2CH_3SO_3$. **A)** and **B)**. Two reversible reductions are observed, which can be assigned to the one electron reduction to the radical **15**$^{\bullet}$ with $E_{1/2}$ (**15**$^{+}$/**15**$^{\bullet}$) = –0.10 V *vs.* SCE **(C)**, and the one-electron reduction of radical **15**$^{\bullet}$ to **15**$^{-}$ with $E_{1/2}$ (**15**$^{\bullet}$/**15**$^{-}$) = –1.07 V *vs.* SCE **(D)**. Both redox potentials are in accordance with reported values for related *N*-methylphenazinium cations (–0.05 V and –1.02 V *vs.* SCE).[7]

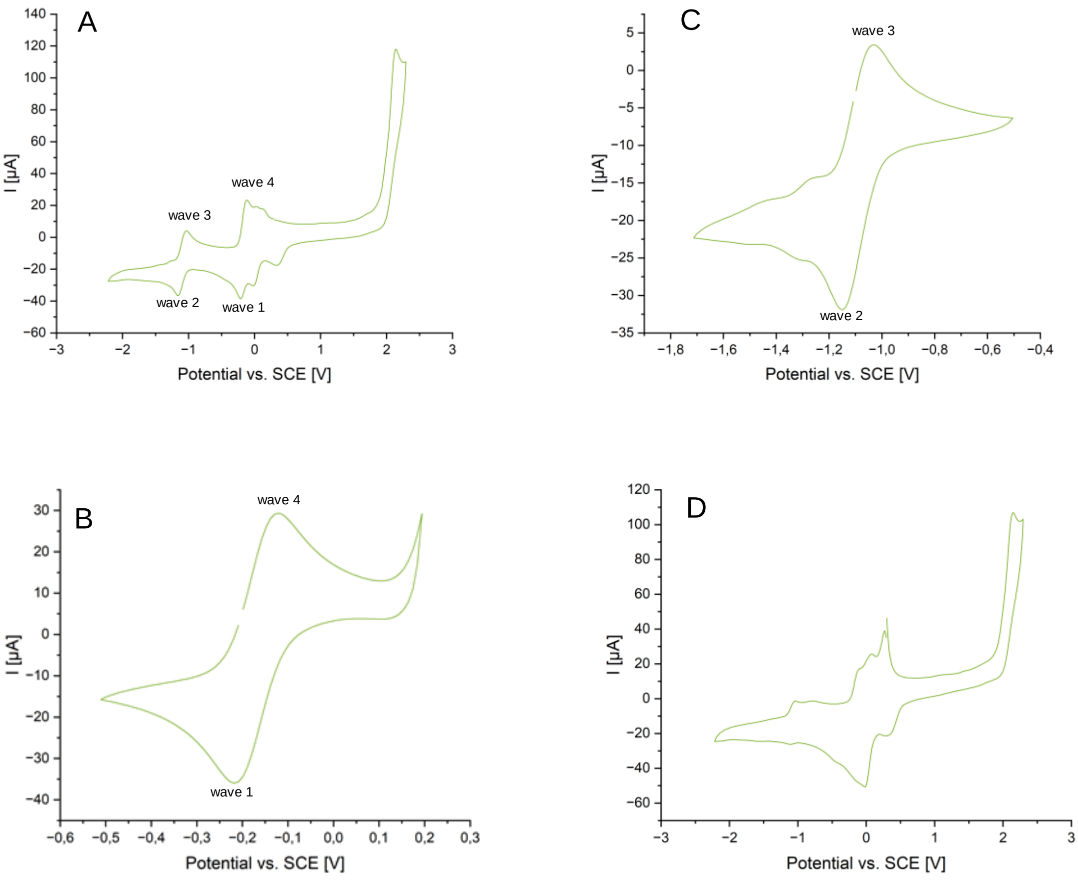

Figure S11. Cyclic voltammogram of compound **16**•OCH_3SO_3. **A)** Two reversible one-electron processes are observed. The first reversible one-electron reduction occurs at a redox potential of $E_{1/2}$ (**16**$^+$/**16**$^•$) = –0.17 V *vs.* SCE **(B)**, while the second reduction of **16**$^•$ to **16**$^–$ is assigned to the redox potential of $E_{1/2}$ (**16**$^•$/**16**$^–$) = –1.10 V *vs.* SCE **(C)**. It has to be noted that the shape of the voltammogram changes after several scans **(D)**. This points towards analyte decomposition and deposition on the surface of the electrodes.[8,9]

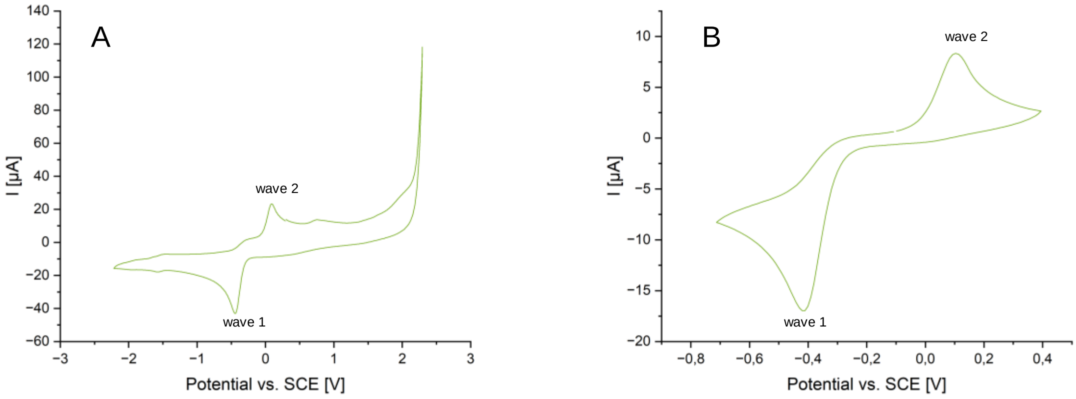

Figure S12. Cyclic voltammogram of compound **20**•OCH_3SO_3. **A)** An irreversible reduction is observed at E_P = –0.43 V *vs.* SCE (wave 1), which is coupled to oxidation wave 2 at E_P = +0.10 V *vs.* SCE **(B)**. The measured peak potential for the reduction agrees with literature reports, where it is assigned to dimer formation after one electron reduction.[10-12]

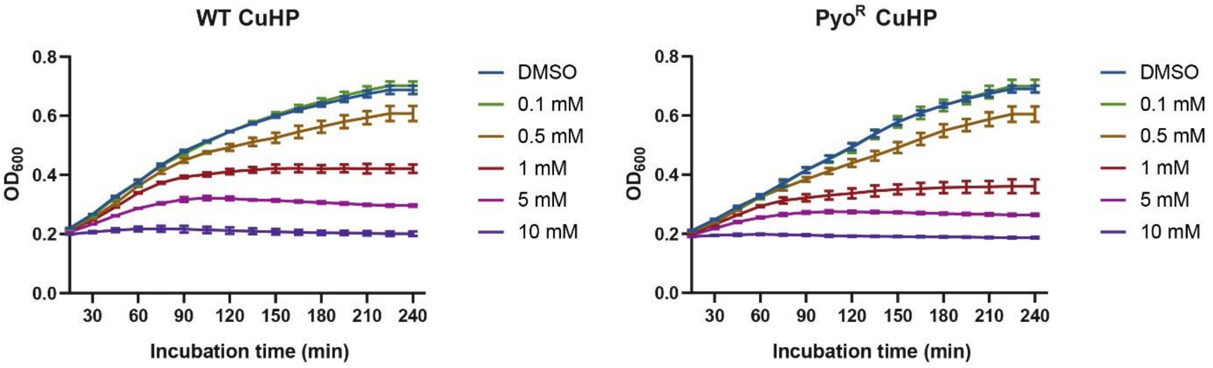

Figure S13. Growth curve of S. aureus ATCC 6341 wild-type and its pyo^R mutant in the presence of varying CuHP concentrations. Experiments were performed in biological triplicates with error bars representing the standard deviations of the replicates, respectively.

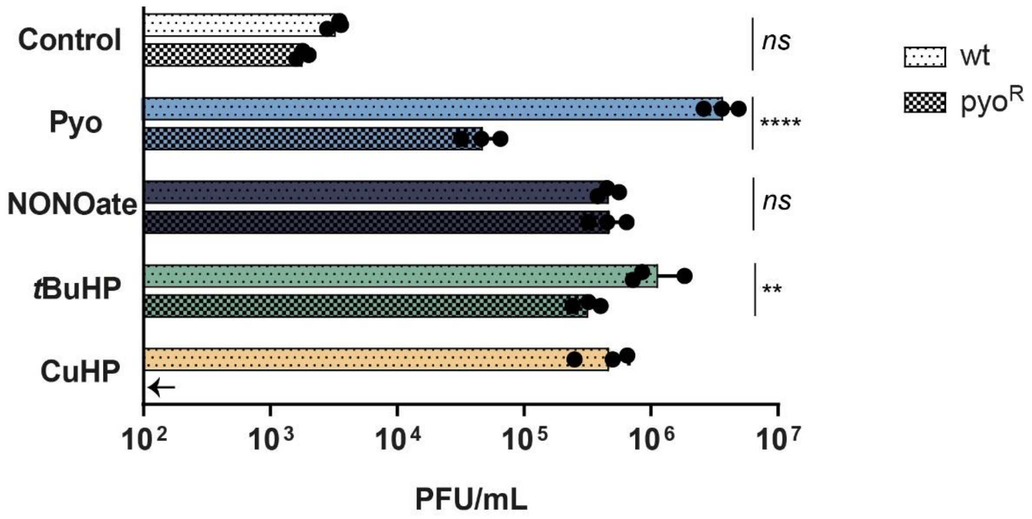

Figure S14. PFU/mL for *S. aureus* ATCC 6341 wild type and the pyo^R mutant upon induction with DEA-NONOate, *t*BuHP and CuHP compared to pyocyanin treatment. Arrow indicates the absence of detectable PFUs. Induction experiments were performed in triplicates and the mean values with the corresponding standard deviations are reported. Statistical significance tests were done with two-way ANOVA, followed by Tukey's test. ****, P<0.0001; **, P<0.01; *ns*, not significant.

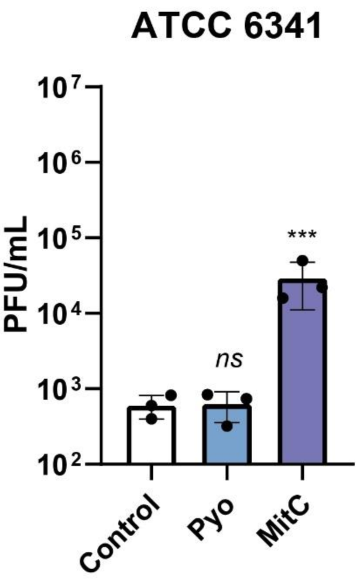

Figure S15. PFU/mL for *S. aureus* ATCC 6341 induced with pyocyanin (25 µM) or mitomycin C (1.5 µM) under anaerobic conditions. Experiments were performed in biological triplicates and the data analysed with one-way ANOVA, followed by Dunnett's test. ***, P<0.001; *ns*, not significant vs control.

13. References

[1] M. Jancheva, T. Böttcher, *J Am Chem Soc* **2021**, *143*, 8344-8351.

[2] M. J. Noto, W. J. Burns, W. N. Beavers, E. P. Skaar, *J. Bacteriol.* **2017***, 199*, e00221–e00217.

[3] D. M. Kasozi, S. Gromer, H. Adler, K. Zocher, S. Rahlfs, S. Wittlin, K. Fritz-Wolf, R. H. Schirmer, K. Becker, *Redox Rep.* **2011***, 16*, 154–165.

[4] V. V. Pavlishchuk, A. W. Addison, *Inorg. Chim. Acta* **2000**, *298*, 97-102.

[5] D. Refardt, *Bacteriophage* **2012**, *2*, 98–104.

[6] A. T. Garrison, Y. Abouelhassan, D. Kallifidas, F. Bai, M. Ukhanova, V. Mai, S. Jin, H. Luesch, R. W. Huigens III, *Angew. Chem. Int. Ed* **2015***, 54*, 14819 –14823.

[7] M. M. Morrison, E. T. Seo, J. K. Howie, D. T. Sawyer, *J. Am. Chem. Soc.* **1978**, *100*, 1, 207–211.

[8] K. J. Lee, B. D. McCarthy, J. L. Dempsey, *Chem. Soc. Rev.* **2019**, *48*, 2927-2945.

[9] D. Sharp, P. Gladstone, R. B. Smith, S. Forsythe, J. Davis, *Bioelectrochem.* **2010**, *77*, 114-119.

[10] S. Ilic, A. Alherz, C. B. Musgrave, K. D. Glusac, *Chem. Commun.* **2019**, *55*, 5583–5586.

[11] P. Hapiot, JL. Moiroux, J. M. Saveant, *J. Am. Chem. Soc.* **1990**, *112*, 1337–1343.

[12] J. Klippenstein, P. Arya, D. D. M. Wayner, *J. Org. Chem*. **1991**, *56*, 6736–6737.

[13] G. J. Sarkis, G. F. Hatfull. In *Mycobacteria Protocols*, T. Parish, N. G. Stoker, Eds.; Humana Press, **1998**,145-173.
